# LncRNA evolution and DNA methylation variation participate in photosynthesis pathways of distinct lineages of *Populus*

**DOI:** 10.48130/FR-2023-0003

**Published:** 2023-02-06

**Authors:** Jiaxuan Zhou, Fangyuan Song, Yuling He, Wenke Zhang, Liang Xiao, Wenjie Lu, Peng Li, Mingyang Quan, Deqiang Zhang, Qingzhang Du

**Affiliations:** 1 State Key Laboratory of Tree Genetics and Breeding, College of Biological Sciences and Technology, Beijing Forestry University, No. 35, Qinghua East Road, Beijing 100083, P. R. China; 2 Key Laboratory of Genetics and Breeding in Forest Trees and Ornamental Plants, Ministry of Education, College of Biological Sciences and Technology, Beijing Forestry University, No. 35, Qinghua East Road, Beijing 100083, P. R. China; 3 The Tree and Ornamental Plant Breeding and Biotechnology Laboratory of National Forestry and Grassland Administration, College of Biological Sciences and Technology, Beijing Forestry University, No. 35, Qinghua East Road, Beijing 100083, P. R. China

**Keywords:** Photosynthesis, Long non-coding RNA, Evolution, DNA methylation, Geographical accessions

## Abstract

During the independent process of evolution in plants, photosynthesis appears to have been under convergent evolution to adapt to specific selection pressure in their geographical regions. However, it is unclear how lncRNA regulation and DNA methylation are involved in the phenotypic convergence in distinct lineages. Here, we present a large-scale comparative study of lncRNA transcription profile and whole-genome bisulfite sequencing (WGBS) data in two unrelated *Populus* species, selected from three relatively overlapping geographical regions. The results indicated that 39.75% lncRNAs of *Populus tomentosa* were shown to have homologous sequences in the 46.99% lncRNA of *Populus simonii*. Evolutionary analysis revealed that lncRNAs showed a rapid gain rate in the *Populus* lineage. Furthermore, co-expression networks in two *Populus* species identified eight lncRNAs that have the potential to simultaneously *cis-* or *trans-*regulate eight photosynthetic-related genes. These photosynthetic lncRNAs and genes were predominantly expressed in accessions from the southern region, indicating a conserved spatial expression in photosynthetic pathways in *Populus*. We also detected that most lncRNA targeted photosynthetic genes hypomethylated in promoter regions of Southern accessions compared with Northern accessions. Geographical DMRs correlated with genetic SNP variations in photosynthetic genes among *Populus* from the three geographic regions, indicating that DNA methylation coordinated with lncRNAs in convergent evolution of photosynthesis in *Populus*. Our results shed light on the evolutionary forces acting on patterns of lncRNA and DNA methylation, and provide a better understanding of the genetic and epigenetic mechanism in photosynthetic convergence evolution.

## INTRODUCTION

Convergent evolution has been extensively reported in cases of humans^[1]^, woody plants^[2]^, cereal crops^[3]^, and Orchidaceae plants^[4]^. Many morphological and physiological traits have been undergoing strong convergent selection, especially in plants. Most convergent traits are not linked to core metabolism^[5]^, but photosynthesis is one of the exceptions^[6]^. Biochemical pathways involved in the capture of atmospheric carbon is more variable than sequestration of light energy. Thus, investigation of genetic and epigenetic regulatory mechanisms canalizing the convergent evolution in photosynthetic pathways, such as non-coding RNAs and DNA methylation is critical for plants' growth and development during long-term local environment adaptation.

In recent years, long non-coding RNAs (lncRNAs) was regarded as one of the important regulators of gene expression of multiple biological processes^[7−9]^. Several studies have shown that lncRNAs involved in the photosynthesis process. For example, one *Arabidopsis* lncRNA *HIDDEN TREASURE 1* (*HID1*) acts through *PHYTOCHROMEINTERACTING FACTOR 3* (*PIF3*), which promotes photomorphogenesis in continuous red light^[10]^. In the anthocyanin-associated coloration pathway, lncRNA *MdLNC610* upregulate the expression level of *MdACO1* by increasing the ethylene production and anthocyanin levels under high-light treatment^[11]^. Moreover, lncRNAs evolve rapidly and are poorly conserved among distantly related species^[12,13]^. LncRNA evolutionary analysis provides great benefit to the understanding of the functions of lncRNAs and the evolution of regulatory networks. The function of ancient lncRNAs may regulate embryonic development and conserved lncRNAs in lncRNA families probably function in many fundamental processes^[14]^. However, how lncRNAs play roles of adaptative evolution in lineage plants, and to what extent can lncRNAs carry similar functions in photosynthetic pathways in plants, remains largely unknown.

The function of lncRNAs in regulating gene expression can be affected by genetic and epigenetic variation^[15−17]^. DNA methylation is one of the vital epigenetic modifications that is widespread in the genome of eukaryotes^[18]^. DNA methylation is heritable during the change of development or affected by environmental conditions. Their adaptive variation may directly evolve through adaptive responses to a changing environment or arise from adaptive genetic variation. Environment-induced epigenetic variation may be limited and restricted to certain regions of the genome during the inheritance^[19]^. The epiallele near the functional gene in the maintainenance of chloroplast structures participate in the regulation of many genes associated with photosynthesis processes^[20]^. Over long timescales, genetic variations affect DNA methylation pattern, and associated with segregating structural variants or with mutations in methyltransferase genes^[21]^. LncRNAs could guide DNA methylation and silence target genes, investigation of the DNA methylation status would enhance the understanding of the regulatory roles of lncRNAs^[22]^. The expression level of lncRNA was also tightly linked with DNA methylation during the plants development and adaptation to the environment^[23]^. Yet, more investigations are still needed to unearth the epigenetic variation underlying photosynthetic pathways to response to local adaptation and its lncRNA relevance in the related species.

In this study, transcriptome analysis was performed to systematically identify lncRNAs and characterize their expression patterns in two unrelated *Populus* species, *P. tomentosa* and *P. simonii*. The convergent emergence or loss of photosynthetic phenotypes may facilitate adaptation to ecologically similar environments. The regulatory roles of the lncRNAs were investigated by co-expression between lncRNAs and their target genes enriched in photosynthetic pathways. Based on the evolutionary analysis of lncRNAs of nine diverse plant species including *P. tomentosa*, *P. simonii*, *Populus trichocarpa*, *Salix purpurea*, *Arabidopsis thaliana*, *Glycine max*, *Oryza sativa*, *Zea mays*, and* Physcomitrella patens*, we identified rapid evolvement even between closely related plants. Also, the potential DNA methylation and adaptive DNA sequences that can be subject to evolutionary divergence as methylation variation correlated with SNP variation. The transcription of lncRNAs target genes is fine-tuned through epigenetic modifications. As a result, photosynthetic genes are representative of adaptive evolution governed by the joint and complementary actions of lncRNAs and epigenetic processes.

## MATERIAL AND METHODS

### Plant materials and phenotypic data measurement

Ten *P. tomentosa* accessions and ten *P. simonii* accessions were collected from their natural population clonal garden in Guan Xian County, Shandong Province, China (36°10′ N, 114°35′ E). The sampling accessions were selected from the Southern (S), Northwestern (NW), and Northeastern (NE) geographical regions according to their natural distribution^[24,25]^. In 2019, tree seedlings were planted with three replicates in the same location using the root segment technique.

The measurement for photosynthetic traits were taken on a Li-COR 6400XT portable photosynthesis system (Lincoln, NE, USA). The leaf chamber conditions were: light intensity 1,000 μmol·m^−2^·s^−1^ PAR and flow 400 μmol·s^−1^. Only mature leaves of each plant were measured. All sampling was measured on clear, sunny days between 09:00 and 11:00 in June, 2019. The measurement was performed using three replications per individual. We measured net photosynthetic rate (Pn, µmol·m^−2^·s^−1^), conductance to H_2_O (Cond, mol·m^−2^·s^−1^), intercellular CO_2_ concentration (Ci, µmol·mol^−1^), and transpiration rate (Trmmol, µmol·m^−2^·s^−1^). Water use efficiency (WUE) was determined by photosynthetic rate over transpiration^[26]^. All measured leaves for each individual were collected, frozen in liquid nitrogen, and stored at −80 °C until use.

### RNA isolation, RNA-sequencing and expression analysis

Total RNAs were isolated from leaves of both *P. tomentosa* and *P. simonii* samples using the Plant Qiagen RNeasy kit which were used for RNA-seq (Methods S1). The clean reads of *P. tomentosa* were mapped to the *P. tomentosa* reference genome, and the clean reads of *P. simonii* were mapped to *P. trichocarpa* reference genome v4.0 (www.phytozome.net) using Hisat2 version2.1.0^[27]^ (Supplemental Table S2). FPKM (fragments per kilobase of transcript per million fragments) values were calculated by Cufflinks v2.1.1^[28]^. The edgeR software package^[29]^ was employed to identify differentially expressed genes (DEGs) between pairs of samples from different geographical regions of *P. tomentosa* and *P. simonii*, respectively, with FDR ≤ 0.05 and fold change ≥ 1.

### Identification of lncRNAs and prediction of their target Protein-coding genes (PCGs)

In this study, we integrated RNA-seq data sets of ten *P. tomentosa* and ten *P. simonii*, respectively. Each transcriptome was assembled separately by StringTie2^[30]^ and merged by gffcompare, while the transcript with FPKM > 0.5, length > 200, coverage > 1 was filtered. Coding Potential Calculator2 (CPC2) software^[31]^, Coding Noncoding Index (CNCI) software^[32]^, and PLEK^[33]^ were used to evaluate the coding potential of the remaining transcripts. All transcripts with CPC2 labeled as 'coding', or CNCI > 0, or PLEK scores > 0 were discarded. Finally, the class code 'u' refers to the long intergenic noncoding RNAs (lincRNAs), class code 'x' refers to long noncoding natural antisense transcripts (lncNAT), class code 'j' refers to the sense transcripts, and class code 'i' refers to the intronic transcripts. Differentially expressed lncRNAs were calculated in the same way as DEGs above. The GC contents of these lncRNAs were calculated with the GEECEE tool in EMBOSS^[34]^.

Homologous transcription of lncRNA between lncRNAs transcripts of *P. tomentosa* and *P. simonii* was performed using the BLASTN software. Alignments with E-value < 1e-5, coverage > 50%, identity > 80% were identified as Conserved lncRNA. Otherwise, lncRNAs were denoted as species-specific lncRNAs.

### Target PCGs prediction of lncRNAs

The potential target genes of lncRNAs were predicted *via* *cis* and *trans* analyses. PCGs around lncRNAs within 10 kb upstream or downstream in genome position were pointed as the potential *cis*-target genes^[35,36]^. The potential *trans*-targets in the *Populus* PCGs database was based on PCGs sequence complementarity and RNA duplex energy predictions. First, protein sequences of lncRNAs target PCGs in *P. tomentosa* and *P. simonii* were used as query sequences in BLASTN with E-value < 1e-5 and identity > 80% to identify homologs. Then RNAplex was used to screen lncRNA–PCGs (duplexes RNAplex -E-60) that exhibited complementary base pairing^[37]^.

### Network construction of co-expressed transcripts

The WGCNA 1.70.3 package in R^[38]^ was used to construct the unsigned co-expression network. One-step network construction and module detection method were adopted in both *P. tomentosa* and *P. simonii* with the following parameters: the minModuleSize was 100, and the cut height was 0.25. The soft power was 5 and 12 in *P. tomentosa* and *P. simonii* networks, respectively. To relate traits to the network, we calculated correlations between module eigengenes and the five photosynthetic traits.

### Data source

FASTA sequences for the lncRNAs from nine plants were downloaded from CANTATAdb2.0 and NCBI. 2,990 lncRNAs of *Populus trichocarpa* from Ye et al.^[39]^, 2003 lncRNAs from *Physcomitrella patens* NCBI annotation (www.ncbi.nlm.nih.gov/genome/?term=Physcomitrella+patens), 3,270 lncRNAs from *Oryza sativa* NCBI annotation (www.ncbi.nlm.nih.gov/genome/?term=Oryza+sativa), 5,355 lncRNAs from *Zea mays* NCBI annotation (www.ncbi.nlm.nih.gov/genome/?term=Zea+mays), the 4,070 predicted lncRNAs of *Salix purpurea* from CANTATdb 2.0 database, 3,365 lncRNAs from *Glycine max* NCBI annotation (www.ncbi.nlm.nih.gov/genome/?term=Glycine+max), and 3,480 lncRNAs from *Arabidopsis thaliana* NCBI annotation (www.ncbi.nlm.nih.gov/genome/?term=Arabidopsis+thaliana).

### Phylogenetic tree construction and inference of the birth, death, and age of lncRNA families

To gain insight into lncRNA evolution in plants, nine plants were used for comparisons. First, the phylogenetic tree was obtained *via* OrthoFinder. Single-copy lncRNAs were aligned using nucleotide sequence by MAFFT, and a species tree was built using IQTREE with the default parameters. r8s was performed to establish an ultrametric tree (chronogram) using species tree rooted with *Physcomitrella patens*. The birth, death, age, and ancestral contents of lncRNA families were assessed *via* COUNT software^[40]^ using Dollo-Parsimony with default settings.

### Whole-genome bisulfite sequencing analysis

Total DNA of ten *P. tomentosa* accessions and ten *P. simonii* was extracted from the collected leaves. DNA extraction was performed using a DNase Plant Mini Kit (Qiagen China, Shanghai, China) for whole-genome bisulfite sequencing analysis. The libraries were sequenced on the Illumina HiSeq 4000, and the sequencing reads were filtered using Trimmomatic^[6]^. Paired-end reads of *P. tomentosa* and *P. simonii* genomes were aligned to *P. tomentosa* and *P. trichocarpa* V4.0 genome respectively using Bismark (version 0.16.1)^[41]^ with the default parameters. Methylation cytosine sites with less than five methylated reads were removed. Integrative Genomics Viewer software^[42]^ was used to visualize the DNA methylation Dataset. MethylKit were used to identified Differentially methylated regions (DMRs)^[43]^ (Methods S2).

### SNP genotype calling and positional association analysis with DNA methylation

A total of ten accessions of *P. tomentosa* and *P. simonii* were sequenced on the Illumina GA II platform with an average depth of 15-fold genome coverage. The clean data were collected by removing low-quality reads (< 10% of nucleotides with quality < Q20). The paired-end data were aligned to the *P. tomentosa* and *P. trichocarpa* V4.0 reference genome using Bowtie 2 software with default parameters^[44]^. Samtools and Genome Analysis Toolkit (GATK) were used to perform single nucleotide polymorphisms (SNPs) calling. Low-quality SNPs that missing data ≥ 20% were filtered. 12,651,394 and 4,996,309 high-quality SNPs were retained for further analysis. To determine the relationship between DMRs and SNPs, we computed a one-sided permutation test between each pair of DMRs and SNPs within 2 kb upstream and downstream of each DMR. A DMR was determined to correlate with SNPs when there are at least three SNPs significant correlates with this DMR (one-sided permutation *p*-value < 0.01).

## RESULTS

### Photosynthetic variation in *P. tomentosa* and *P. simonii* accessions

**Figure 1:**
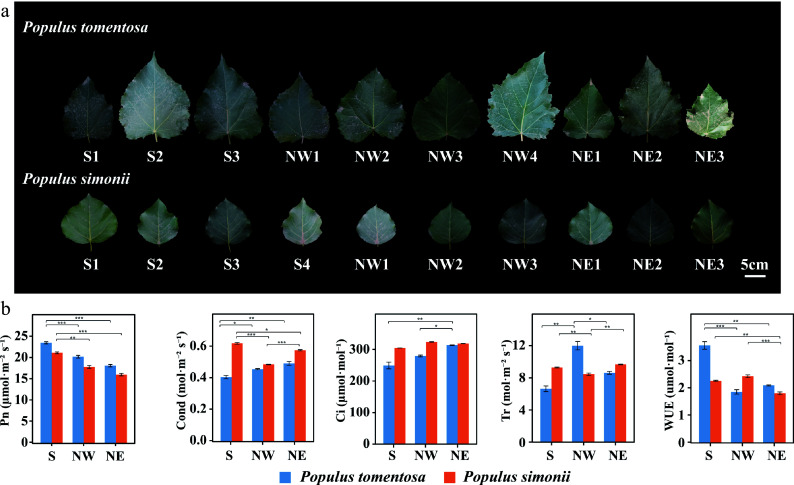
Morphological and photosynthetic variation of *Populus tomentosa* and *Populus simonii* from different geographical regions. (a) Leaf size and shape of *P. tomentosa* and *P. simonii* from the Southern geographical region (S), the Northwestern geographical region, and the Northeastern geographical region (NE). Numbers represent accession number in its geographical region. Scale bar, 5 cm. (b) Photosynthetic traits of *P. tomentosa* (blue) and *P. simonii* (orange) from three geographical regions. Photosynthetic traits include net photosynthetic rate (Pn), conductance to H_2_O (Cond), intercellular CO_2_ (Ci), transpiration rate (Tr), and water use efficiency (WUE). Data represent means ± SE. *, *p* < 0.05; **, *p* < 0.01; ***, *p* < 0.001.

The shape of leaves were associated with photosynthetic abilities of plants and probably contribute to photosynthetic differences of different species^[45−47]^. *P. tomentosa* and *P. simonii* accessions displayed considerable variation in size and shape of leaves (Fig. 1a). As there was considerable macroscopic variation in leaf characteristics, we next investigated whether differences observed in features affected photosynthetic performance. Interestingly, there were statistically significant differences in net photosynthetic rate (*P* = 2.18 × 10^−4^), conductance to H_2_O (*P* = 1.77 × 10^−3^), and intercellular CO_2_ concentration (*P* = 4.0 × 10^−3^) among two *Populus*. For all accessions, photosynthetic traits varied greatly especially in *P. tomentosa*, with coefficients of variation (CV) values ranging from 9.48% (Conductance to H_2_O) to 38.79% (Water use efficiency). Additionally, all five phenotypic traits showed significant differentiation among the geographical regions of both *P. tomentosa* and *P. simonii* (*P* < 0.05, post-test by LSD) (Supplemental Table S1). For example, the net photosynthetic rate of accessions from the Southern region in both *Populus* was significantly higher than those from the Northern regions (Fig. 1b). This showed that photosynthetic variation between accessions from geographical regions in *P. tomentosa* and *P. simonii* may be partly due to the underlying selective pressure in their environments^[48]^. Therefore, differences in transcripts expression and regulatory networks are critical to determining interspecific and intraspecific phenotypic variation.

### Genome-wide identification, characterization, and expression profile of lncRNAs in *P. tomentosa* and *P. simonii* accessions

**Figure 2:**
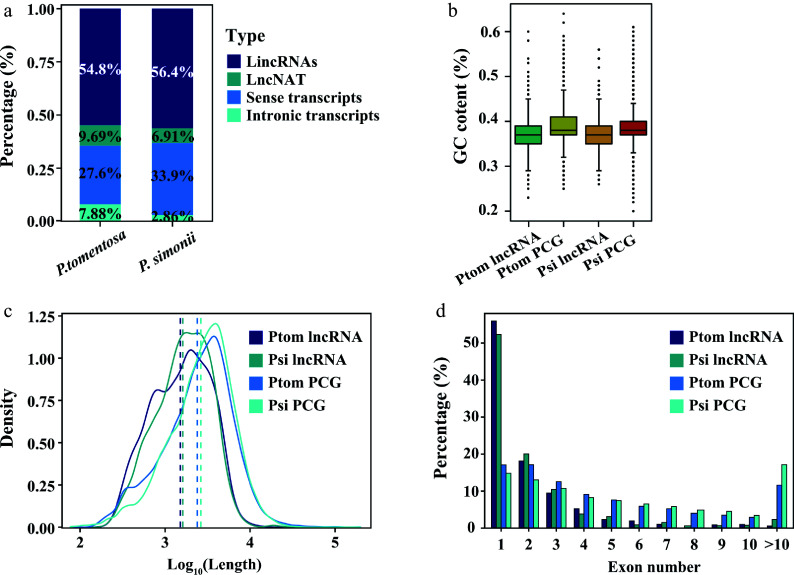
Identification and characterization of lncRNAs in two *Populus* species. (a) Percentage distribution of different classifications of the total lncRNAs in *Populus tomentosa* and *Populus simonii*. (b) The GC content of lncRNAs and protein-coding genes (PCGs) in *P. tomentosa* and *P. simonii*. (c) Length density distributions of lncRNAs and PCGs. The x-axis indicates the log10-transformed sequence length and the y-axis indicates the density value. (d) Percentage distribution of exon numbers for PCGs and lncRNAs.

To obtain a comprehensive profile of lncRNAs in different *Populus* species, we assembled transcriptome using the strand-specific RNA-seq data from ten *P. tomentosa* accessions and ten *P. simonii* accessions. In total, we identified a total of 1,600 and 1,013 high-confidence lncRNAs in *P. tomentosa* and *P. simonii*, respectively (Data S1, S2). Four classes of lncRNAs were identified, and the majority of them were long intergenic noncoding RNAs (lincRNAs) and long noncod natural antisense transcripts (lncNATs) in both species (Fig. 2a). We then investigated the characters and expression profile of these lncRNAs between two species. The lncRNAs are unevenly distributed across the 19 chromosomes of both* Populus* species, and there was no difference in GC content between *P. tomentosa* (37.13%) and *P. simonii* (37.35%) for lncRNA (Fig. 2b). According to genomic locations, the lncRNAs of *P. tomentosa* range in length from 230 to 13,266 nucleotides(nt), with a median length of 2,009 nt that is significantly shorter than the median length (2,255 nt) of *P. simonii* (Fig. 2c). On average, the lncRNAs of *P. simonii* contain a significantly fewer exons than the *P. tomentosa* lncRNAs. As expected, most of the lncRNAs comprised fewer exons (> 50% consist of one exon) than PCGs (Fig. 2d).

### The evolution and expression of lncRNAs with intraspecific variation between *Populus* species

**Figure 3:**
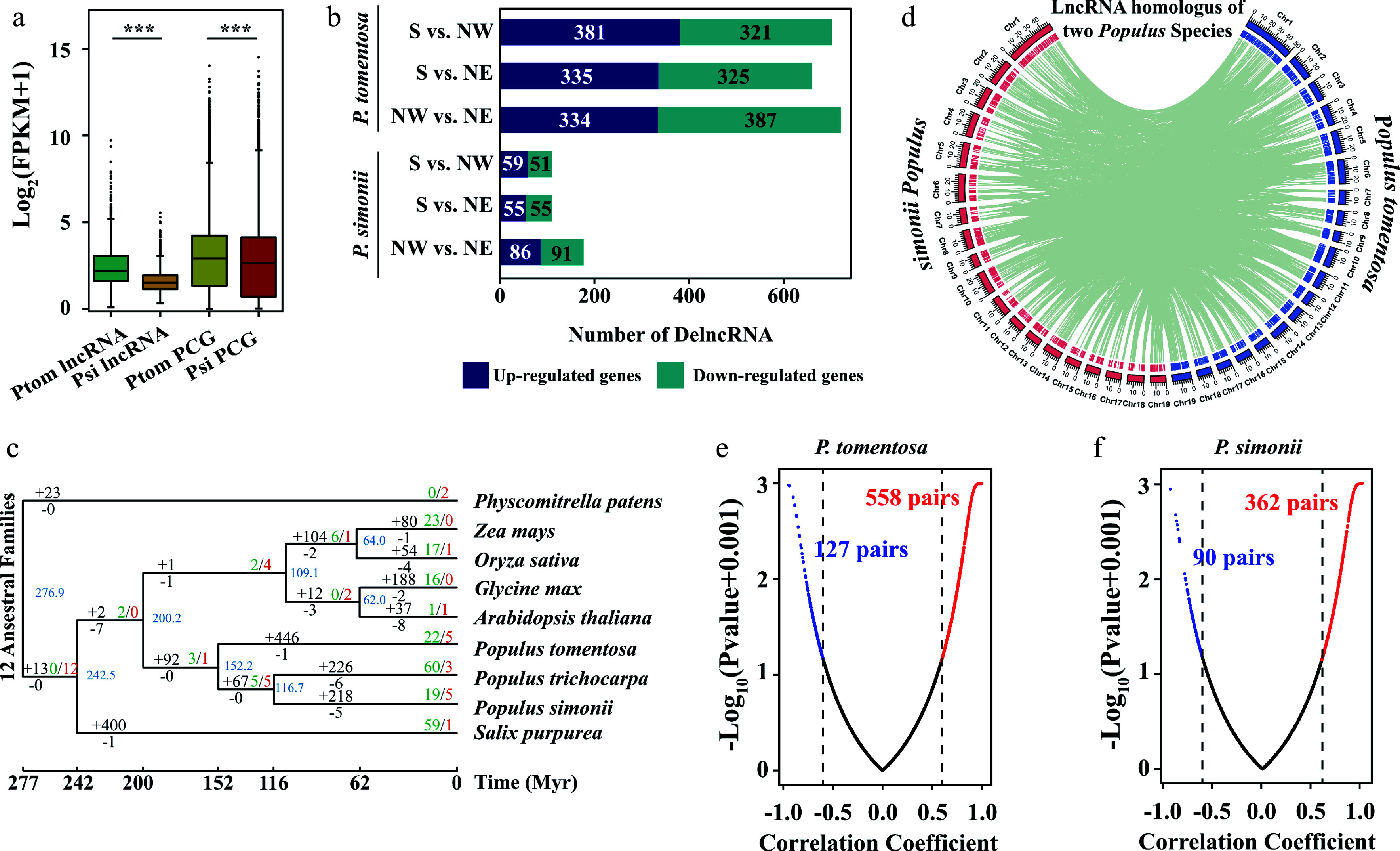
Expression profiles and evolution of lncRNAs in *Populus tomentosa* and *Populus simonii*. (a) Box plot of expression levels of lncRNAs and protein-coding genes (PCGs) in *P. tomentosa* and *P. simonii*. Student t-test was used to calculate the *p*-value. *** *p* < 0.001. (b) The numbers of differentially expressed (DE) lncRNAs in *P. tomentosa* and *P. simonii* between accessions from the Southern geographical region and the Northwestern geographical region, the Southern geographical region and the Northeastern geographical region, the Northwestern geographical region and the Northeastern geographical region. (c) Phylogenetic tree and number of gene families displaying expansion (green) and contraction (red) among nine plant species. Branch lengths reflect evolutionary divergence times in million years (Myrs) inferred from timetrees. Numbers of gained (+) and lost (−) lncRNA families Myr^–1^ (in red) are indicated next to each branch. (d) The distribution of chromosomes (outer) and lncRNAs (inner) in *P. tomentosa* (blue) and* P. simonii* (red). The green lines in the inner rings show lnRNAs that were homoeologous in two *Populus* lineages. (e), (f) Scatter plots of Pearson correlation coefficient and *p*-value between the expressions of the lncRNAs and their target PCGs in* P. tomentosa* and *P. simonii*. The lncRNA-mRNA with correlation coefficient ≥ 0.6 (red) or ≤ −0.6 (blue), and *p*-value ≤ 0.05 are considered positive or negative pairs. For screen visualization,* p*-value were minus log10 transformed after a constant value (0.001) was added.

To evaluate lncRNA differences between *Populus* species and intraspecific variation, we analyzed the lncRNAs expression of each accession of both *Populus* species. The expression level of the lncRNAs from both species was lower than for the PCGs^[28]^ (Fig. 3a). The overall expression levels of lncRNAs in *P. simonii* were lower than that of *P. tomentosa*. We next identified the differentially expressed lncRNAs and PCGs between geographical regions in both species (Data S3−S6). Intriguingly, differentially expressed lncRNAs from *P. tomentosa* (41.25%−45.06%) and *P. simonii* (10.86%−17.47%) occupied a large proportion in their total lncRNAs, but PCGs had a lower expression variation ratio among geographical regions (*P. tomentosa*, 8.18%−12.20%; *P. simonii*, 3.88%−7.36%) (Fig. 3b).

LncRNAs are highly diverged at the nucleotide level among plant species but may have high sequence conservation at the intraspecies and interspecies levels. lncRNA orthologous pairs were identified through reciprocal best hits, and they were connected using the single-linkage clustering method to construct lncRNA families. The phylogenetic tree revealed that the evolution of the lncRNAs spaned around 277 Myrs (million years). We identified 1,033 lncRNA families with a total of 3,775 conserved lnRNAs. We then sought to investigate the birth and death rates and the ancestor lncRNA families during the plant evolution. Among these lncRNA families, the number of lncRNA families increased from 12 ancestral families to 35−557 families in all the plant species (Fig. 3c). Notably, terminal branches gained more families than internal branches, particularly in Salicaceae trees. The highest net gain rates in recent terminal branches in Salicaceae trees ranging from 1.65 to 5.02 families Myr^−1^, indicating a high rate of novel lncRNA families in forest trees. In addition, 482 (47.58%), 669 (22.39%), and 746 (18.33%) lncRNAs of *P. tomentosa* were found to be conservation in *P. simonii*, *P. trichocarpa*, and *Salix purpurea*, respectively. These results suggested that most lncRNAs were conserved between *P. tomentosa* and *P. simonii* despite rapid gene fractionation.

To explore whether lncRNAs contribute to evolutionary pressures on plant photosynthesis, we compared conserved lncRNAs with species-specific lncRNAs in two *Populus* species. Using the reciprocal align features of BLASTN, there are 3,305 homoeologous lncRNA pairs between *P. tomentosa* and *P. simonii* (Fig. 3d; Data S7). We found that 39.75% lncRNA of *P. tomentosa* had homologous copies in the 46.99% lncRNAs of *P. simonii*. These results suggested that the vast majority of lncRNAs were species-specific or limited to closely related species.

To identify genes potentially regulated by lncRNAs and the potential effects of lncRNAs, we identified 685 and 452 lncRNA-PCGs pairs in *P. tomentosa* and *P. simonii*, respectively. Expression analysis on the lncRNAs-PCGs pairs showed that 81.46% and 80.09% of them have a positive correlation (|r_*p*_| ≥ 0.6, *P* < 0.05) in two *Populus* species (Fig. 3e, f). On average, the r_*p*_ between expression of *P. tomentosa* lncRNAs and their targets PCGs (0.48) was higher than that between adjacent PCGs pairs (0.38), which was similar to lncRNA-random PCG pairs (0.39). These correlations were much stronger than those of PCG-random PCG pairs (0.18) (Supplemental Fig. S1a). Moreover, the ratio of extreme expression correlation (|r_*p*_| > 0.8) for lncRNA-PCG pairs (25.84%) was higher than those of lncRNA-random PCG pairs (19.09%) and PCG-random PCG pairs (23.54%) (Supplemental Table S2). Similar results were observed in *P. simonii* (Supplemental Fig. S1b).

### LncRNA participates in the regulation network of photosynthetic variation between *P. tomentosa* and *P. simonii*

**Figure 4:**
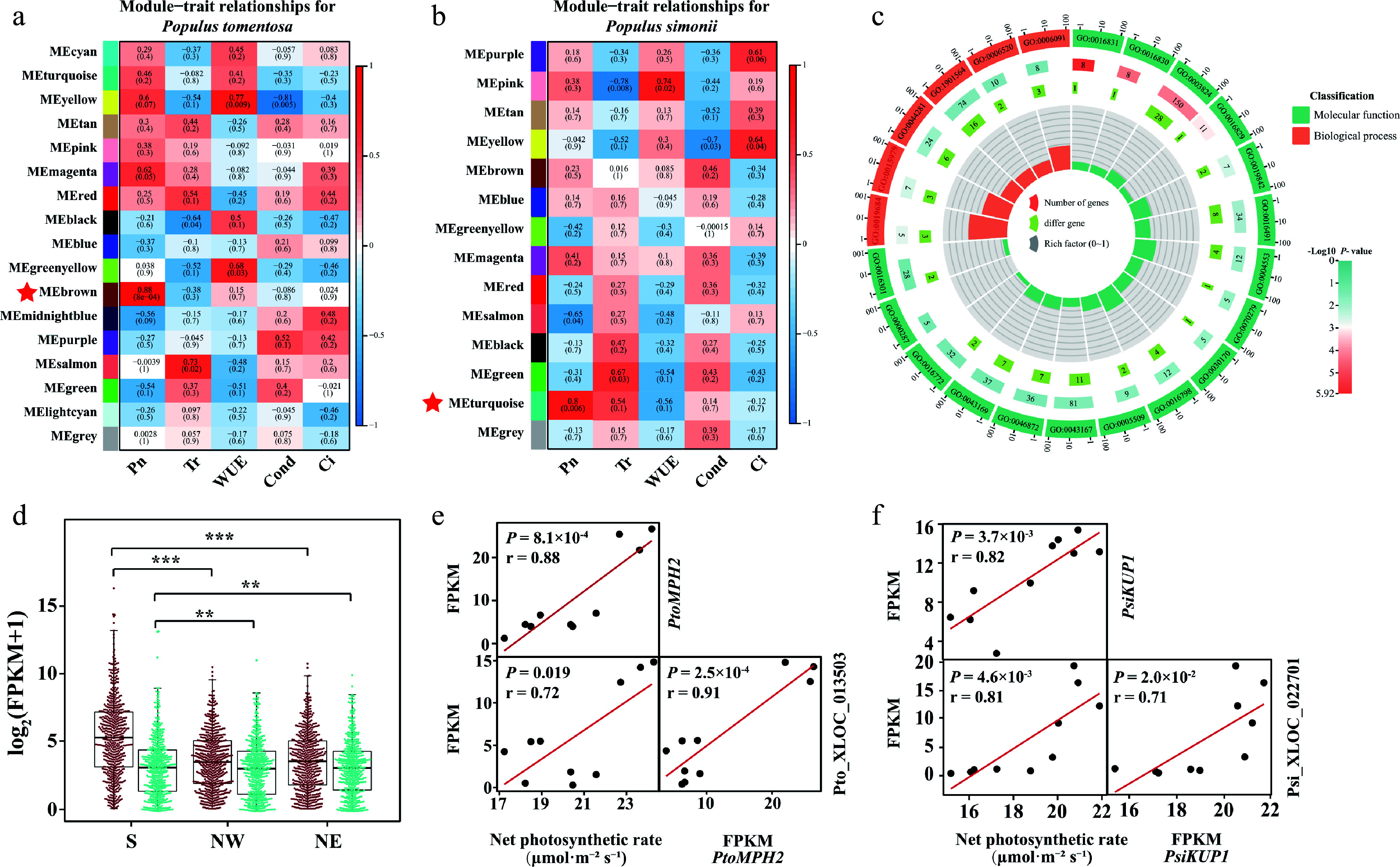
Photosynthetic-associated modules in *Populus tomentosa* and *Populus simonii*. Statistical analysis of module–trait correlations in (a) *P. tomentosa* and (b) *P. simonii*. The rows and columns indicate the modules and traits, respectively. Cells are colored from blue to red according to the Pearson correlation coefficient in parentheses, and the star-marked cells indicate the highest significant association between the trait and its corresponding module. (c) Circos plot shows the enrichment and differentially expressed genes in each ontology of Module Brown in *P. tomentosa*. From the outer to the inner circle, is gene ontology (GO) id, number of genes and *P* -value, number of differentially expressed genes, and enrichment factors. (d) Box plot and beewarm plot of expression levels of lncRNAs and protein-coding genes (PCGs) in Module Brown from *P. tomentosa* and Module turquoise from *P. simonii*. Student t-test was used to calculate the *p*-value. *** *p* < 0.001, ** p < 0.01. (e) Scatter plots show correlation between the expression of *PtoMPH2* and net photosynthetic rate, expression of Pto_XLOC_013503 and net photosynthetic rate, and between expression of *PtoMPH2* and Pto_XLOC_013503. Pto_XLOC_013503 positively regulate a chloroplast thylakoid lumen protein, *PtoMPH2*. *r*, Pearson correlation coefficient; *p*, significance of the correlation between trait and gene expression. (e) Scatter plots show correlation between the expression of *PtoMPH2* and net photosynthetic rate, expression of Pto_XLOC_013503 and net photosynthetic rate, and between expression of *PtoMPH2* and Pto_XLOC_013503. *r*, Pearson correlation coefficient; *p*, significance of the correlation between trait and gene expression. (f) Scatter plots show correlation between the expression of *PtoKUP1* and net photosynthetic rate, expression of Pto_XLOC_022701 and net photosynthetic rate, and between expression of *PtoKUP1* and Pto_XLOC_022701. r, Pearson correlation coefficient; P, significance of the correlation between trait and gene expression.

Although photosynthesis is one of the basic biochemical reactions of plants, they exhibit dramatic differences in multiple characteristics. Differences in gene expression and regulatory networks are critical for determining photosynthesis traits. We performed a weighted gene co-expression network analysis (WGCNA) on PCGs and lncRNAs. Accordingly, we obtained 16 and 13 distinctly expressed modules in* P. tomentosa* and *P. simonii*, respectively (Fig. 4a, b). The modules closely related to photosynthetic traits and GO term were of particular interest in this study. 'Photosynthesis, light reaction' (GO:0019684) and 'photosynthesis' (GO:0015979) GO terms were enriched in the subset of PCGs in MEbrown of *P. tomentosa* network and MEturquoise of *P. simonii* network (Fig. 4c; Supplemental Fig. S2; Data S8, S9). In *P. tomentosa*, the module 'Brown' comprised transcripts that were restrained in Northern regions (Fig. 4d). For seven genes enriched in photosynthetic terms, four hub genes including *PtoPPL1*, *PtoLHCA1*, *PtoPnsb4*, and *PtoMPH2* were highlighted in the network due to their high eigengene connectivity (Supplemental Table S3). Among those hubgenes, we noted a *cis*-regulated and three *trans*-regulated lncRNAs, which were differentially expressed between distinct geographical regions (Fig 4e). In *P. simonii*, expression of lncRNAs and PCGs in module 'turquoise' was also highly expressed in the Southern geographical region. Three of the nine photosynthetic-enriched genes were highlighted as high eigengene connectivity, including *PsiLHCB7*, *PsiPSBR*, and *PsiKUP1* (Supplemental Table S3). Gene *PsiKUP1*, a photosynthetic protein pathway gene that encodes a high-affinity potassium transporter, exhibited the highest expressed level in the S region as compared with NW and NE regions (Fig 4f). Meanwhile, we demonstrated that Psi_XLOC_022701 in the S region was more than two times higher expression than those accesions in the N region, indicating that Psi_XLOC_022701 may *trans*-regulate *PsiKUP1*.

### Similar expression patterns of homologous lncRNAs in *P. tomentosa* and *P. simonii*

**Figure 5:**
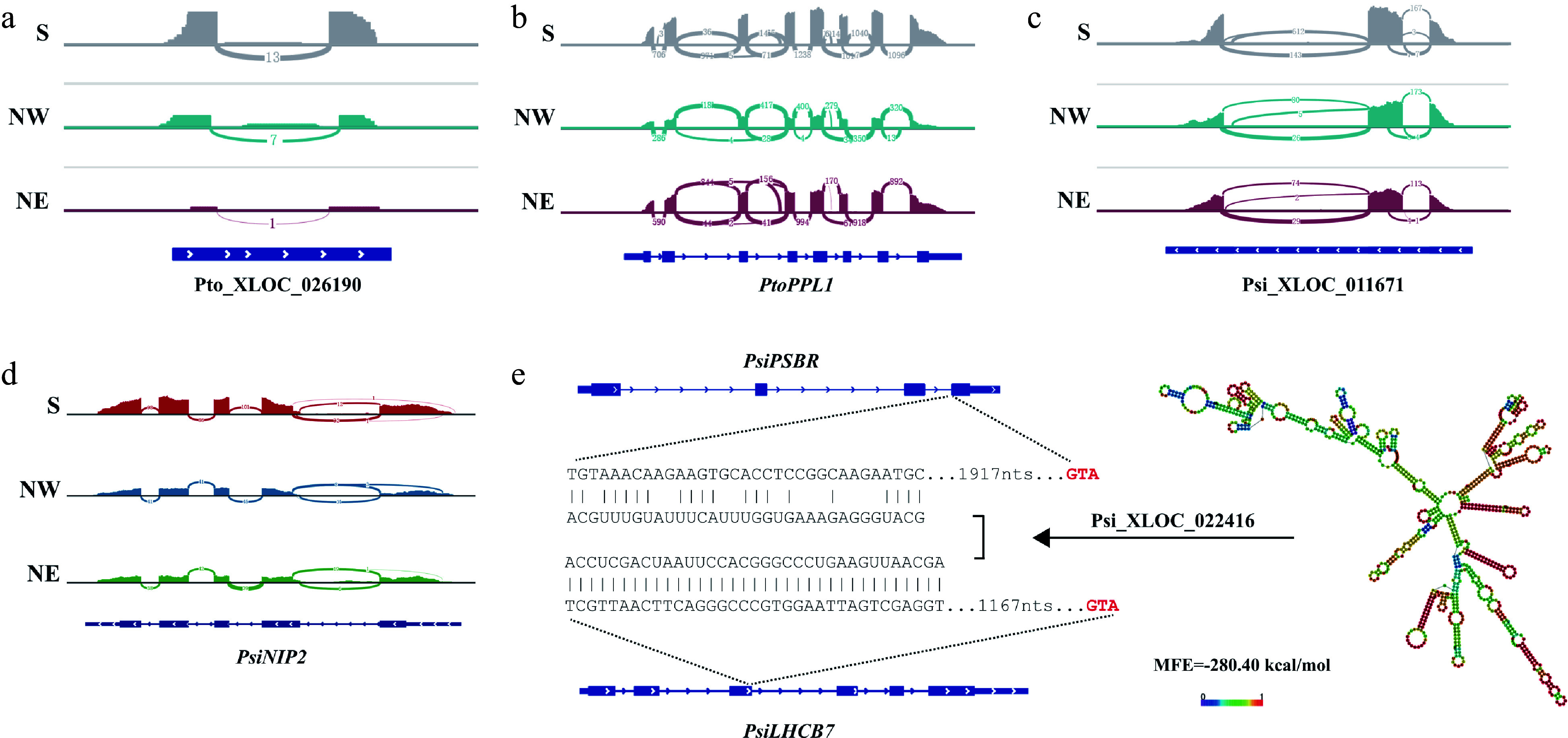
LncRNAs have positive regulatory roles for photosynthetic genes and working model of Psi_XLOC_022416. (a) Pto-XLOC_026190 and its regulatory gene (b) *PtoPPL1*. The RNA-seq coverage of the genes was extracted from the leaf transcriptomes of *P. tomentosa* accessions from the Southern geographical region (S), the Northwestern geographical region (NW), and the Northeastern geographical region (NE), and the numbers in the panel indicate the mapping read counts of junction reads or exonic reads. (c) Psi-XLOC_011671 and its regulatory gene (d) *PtoNIP1*. The RNA-seq coverage of the genes was extracted from the leaf transcriptomes of *P. simonii* accessions from the Southern geographical region (S), the Northwestern geographical region (NW), and the Northeastern geographical region (NE), and the numbers in the panel indicate the mapping read counts of junction reads or exonic reads. (e) A proposed working model of Psi_XLOC_022416 in regulating *PsiPSBR* and *PsiLHCB7* expression to modulate *Populus* photosynthetic variation in different geographical regions.

Conserved lncRNAs across species can provide further information to demonstrate their possible functions and the processes^[49,50]^. Combining the results of co-expression analysis and the origination of lncRNAs in two poplars, we were able to update the putative interspecies and intraspecies expression of the lncRNAs involved in the photosynthesis pathway (Data S3). For *P. tomentosa*, Pto_XLOC_026190 was highly expressed in the S region (Fig. 5a) and *trans*-regulated *Ptom.010.01955* (facilitates the assembly of the photosystem II supercomplexes, *PtoPPL1*) (Fig. 5b). The regional differentiation was potentially similar to the patterns of Psi_XLOC_011671 homologous lncRNA (Fig. 5c), which positively regulated *Potri.007G061400* (encoding a light stimulus response gene, *PsiNIP2*) (Fig. 5d). Moreover, another Pto_XLOC_001831 that contained two homologous lncRNA pairs in *P. simonii* was predicted to target *Ptom.012.00615* (encoding a subunit of the chloroplast NAD(P)H dehydrogenase complex, which involved in PSI cyclic electron transport, *PtoPnsB4*). We found that *PtoPnsB4* was highly expressed in the NE region for *P. tomentosa*. Additionally, homologous lncRNAs of Pto_XLOC_001831 in *P. simonii* also targeted two photosynthetic-related genes including *PsiPSBR* and *PsiLHCB7*. This can be inferred that Pto_XLOC_001831 showed high similarity with Psi_XLOC_022416 and may have functions in the regulatory network of photosynthesis. Both lncRNAs were highly expressed in the S region compared to the NE region. These phenomena indicated that Pto_XLOC_001831 was an evolutionarily more important lncRNA than Pto_XLOC_026190 in *P. tomentosa* (Fig. 5e). It also suggested that the regulatory module is highly conserved across different poplar species and may be functionally maintained.

### The differences in DNA methylation between three geographical regions in *P. tomentosa* and *P. simonii*

Many studies have found that DNA methylation level correlated with the expression level of PCGs. The covariation of DNA methylation and other genetic factors causes phenotypic variation during plants' growth and development^[51−53]^. To investigate the divergence of genomic DNA methylation on photosynthetic variation, we analyzed the difference in methylation patterns associated with photosynthetic variations in *P. tomentosa* and *P. simonii* accessions. More than 100 million cytosines were sequenced in each sample, a number sufficient for further analysis (Supplemental Table S4). *P. tomentosa* displayed an average of 66.31%, 46.85%, 3.75% methylation in CG, CHG, and CHH contexts, respectively. Correspondingly, *P. simonii* presented a mean level of 40.34%, 32.55%, and 4.81% methylation in CG, CHG, and CHH contexts, respectively. We detected distinct differences in CV among three contexts, with the lowest diversity for CG context and the highest for CHH context (Supplemental Table S5). Accessions from the same geographical regions were often closely correlated, especially in CG context (Supplemental Fig. S3a, S3b). These results indicated that both *Populus* accessions possessed significant methylation variability and could contribute to trait variation.

**Figure 6:**
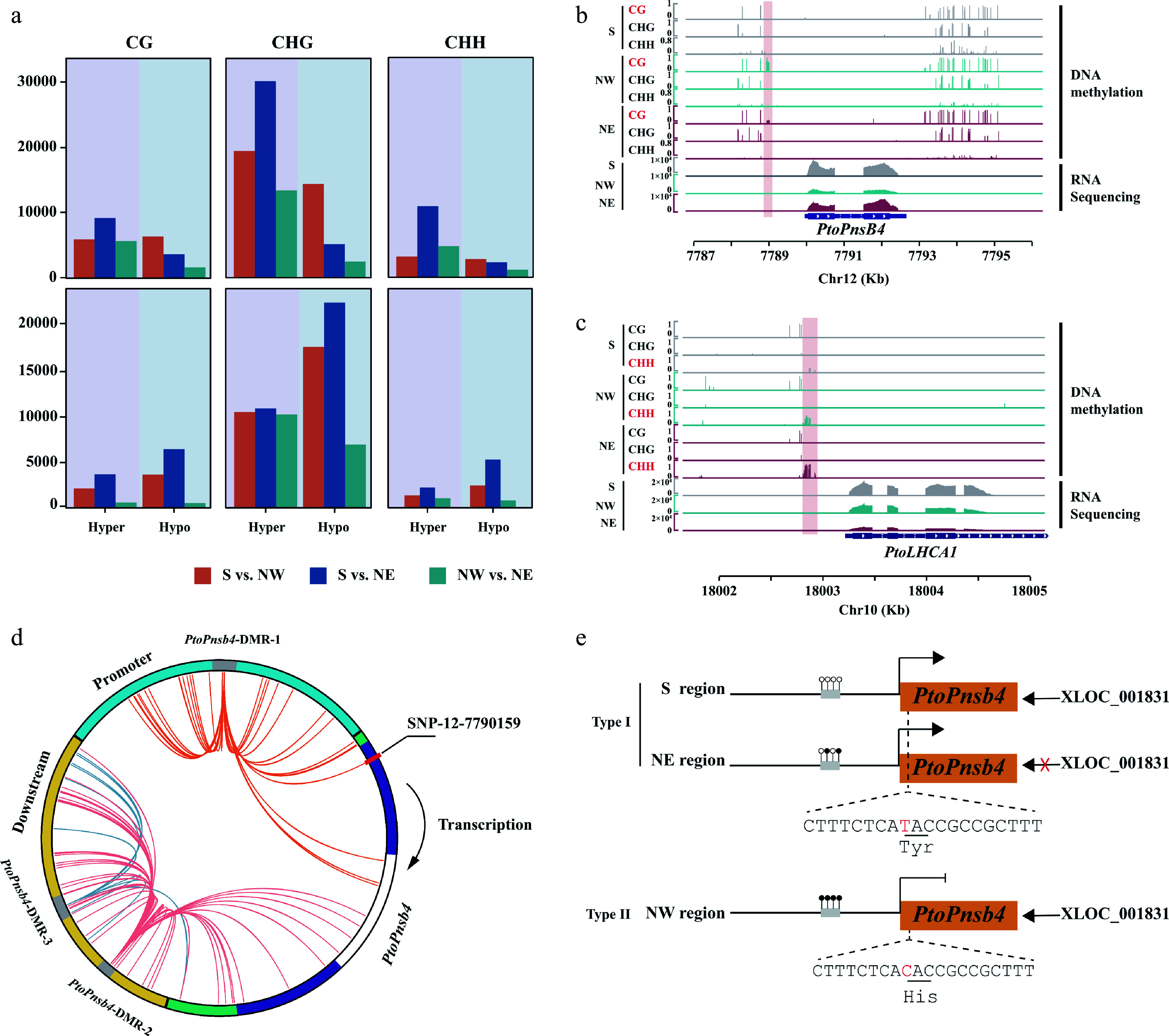
Geographical variation of DNA methylation and genetic variation affect the function of lncRNA. (a) Count of hyper and hypo differentially methylated regions (DMRs) in *P. tomentosa* and *P. simonii* from three geographical regions in three contexts. (b), (c) Integrative Genomics Viewer plots of WGBS tracks in accessions from the Southern geographical region (S), the Northwestern geographical region (NW), and the Northeastern geographical region (NE), as well as RNA-seq tracks over (b) *PtoPnsB4* and (c) *PtoLHCA1*. The position of the DMRs is indicated by the pink shadow area. (d) Circos plot representing interaction DMR-SNP pairs. The circles show the structure of gene *PtoPnsB4*. Blue, green, purple, white, and yellow arcs represent promoter, untranslated region (UTR), exon, intron, and downstream region, respectively. Interior lines represent the pairwise interactions of DMR-SNP pairwise. Orange, red, and blue lines indicate intra-gene interactions of different DMRs between DMR-SNP pairs. (e) A proposed working model that DNA methylation variation participates in the regulation of Pto_XLOC_001831that modulates *Populus* convergence evolution of photosynthesis.

We identified DMRs to further investigate the differences in DNA methylation between three geographical regions in *P. tomentosa* and *P. simonii*. Our analysis identified 51,892, 61,161, and 28,981 DMRs in S *vs*. NW, S *vs*.NE, and NW *vs*. NE in *P. tomentosa*, respectively (Supplemental Table S6). Hypo-DMRs accounted for 54.82%−82.04% of the total DMRs. Similarly, for *P. simonii*, 23,840 and 34,400 hyper-DMRs were found in NW and NE regions when compared with the accessions from S region, suggesting that the lower methylation in Southern regions in both *P. tomentosa* and *P. simonii* (Fig. 6a). It is noteworthy that, despite DMRs in intergenic regions, 16.05% and 16.65% of DMRs occurred in promoters, suggesting that geographical regions might affect DNA methylation within promoter regions (Supplemental Fig. S4a).

### Division of *Populus* photosynthesis involved in genomic DNA methylation patterns

Based on the presence of lncRNAs and genes with DMRs (designated as differentially methylated genes, DMGs), we identified lncRNA targets that were related to DNA methylation variation. Abundant DMGs (70.00% and 52.40%) were identified in the total lncRNA target genes in *P. tomentosa* and *P. simonii* (Supplemental Fig. S4b), including 464 and 255 DEGs differentially expressed between geographical regions (Supplemental Fig. S4c). To further investigate whether the balance between DNA methylation and lncRNAs is responsible for the natural variation of photosynthetic traits, we investigated the expression and the methylation patterns among different geographical regions of two species. Our study found several lncRNA-targeted DMGs that hypomethylated in the promoter regions of southern accessions (Supplemental Table S7).

To explore the genetic variation and DNA methylation that affect the function of lncRNA in regulating its target genes, we further analyzed the relationship between DMRs and SNP in *P. tomentosa* and *P. simonii* (Supplemental Table S8; Data S10, S11). We identified 339 strong correlated pairs (*P* < 0.01, r^2^ > 0.1) between DMR and SNPs range from 2 to 102 in *P. tomentosa* (Supplemental Fig. S5a). Among these signals, a large proportion of SNPs 60.5% were identified in flanking regions and intergenic regions, only 24.21% of correlated SNPs were from promoter sequences and 9.47% from exon sequences. In *P.simonii*, we found one DMR correlated with three SNPs distributed in the intron and promoter (Supplemental Fig. S5b). We detected a regulatory model that showed the function of DNA methylation and genetic variation affecting the effect of lncRNAs on gene expression. For example, Pto_XLOC_001831 was higher expression in S regions compared with NE, but was not differentially expressed in NW region. The promoter regions of its target, *PtoPnsB4*, showed that the accessions from the NE region had higher methylation levels than the accessions from the S. This hypermethylation in NE accessions inhibited the gene expression in *PtoPnsB4* (Fig. 6b). Two CG DMRs in promoter and downstream flanking regions of *PtoPnsB4* are strongly associated with one and three SNPs in the exon region (Fig. 6d). Interestingly, SNP-12-7790159 (TT) correlated with hypo-methylated DMR in the promoter region in S and NE geographical regions. In comparison to the accessions from the NW, SNP-12-7790159 caused a missense variant resulted in the substitution of Tyr to His. Missense variant in gene transcript together with promoter DNA methylation inhibited the function of Pto_XLOC_001831 of gene and caused a lower expression level in *PtoPnsB4* in accessions from NW regions (Fig. 6e). In addition, the homologue of *PtoLHCA1* in *P. simonii* was hypermethylated in the NE region compared with the S region in CHH context (Fig. 6c). This homologue encodes a component of the light-harvesting complex associated with photosystem I. These results indicated a general coherence of interspecific genetic and epigenetic modification in photosynthetic pathways in two *Populus* species.

## DISCUSSION

### Evolution of lncRNA between two closely related *Populus* species

Increasing numbers of progress have been made in elucidating important roles of plant non-coding RNAs due to their extensive abundance. LncRNA has evolved in multiple molecular mechanisms to survive abiotic stress, such as water stress^[54]^, temperature extremities^[55]^, salinity^[39]^, and heavy metal toxicity^[56]^, etc. This study covered two *Populus* species, *P. tomentosa* and *P. simonii*, representing *Populus* sections white poplar and Tacamahaca Spach, which have similar natural geographical distribution in China. Compared with PCGs, lncRNAs had fewer exons, shorter mean lengths of exons, and were less abundantly expressed across the conditions in two species (Fig. 2c, d), suggesting a similar characteristic among two *Populus* species. Furthermore, plants evolved different lncRNAs expression abundance in response to distinct geographical climates. PCGs and lncRNA pairs have shown a significant contribution of lncRNA in a strong regulatory manner on gene expression^[49,55]^.

It is long been confusing on the evolutionary conservation of lncRNAs, their high levels of sequence divergence make them hard to study. In stark contrast to PCGs, only a small portion of lncRNA sequences (1.8%−52.6%) is conserved across nine species. LncRNAs in Salicaceae trees lack known orthologs in species outside of monocot plants, indicating poor conservation of lncRNAs^[57,58]^. We use three closely related *Populus* species *P. tomentosa*, *P. simonii*, and *P. tritrocarpa* to minimize the effects of genomic sequence divergence. LncRNAs are more frequently gained than lost, and the highest net gain rate was identified in the recent terminal *Populus* species. These results suggested that lncRNA transcription evolved extremely rapidly between closely related plants. The transience of intergenic lncRNA transcription is mirrored by changes to selective pressures acting on their sequences.

### The regulatory network of photosynthetic pattern involving lncRNA and DNA methylation in *Populus*

Forest trees experienced photosynthetic divergence as a direct response to landscape processes and heterogeneity of habitat. The threshold of temperature and limitation of precipitation may vary substantially with local environmental conditions, which leads to heterogeneous responses in tree biological adaptation of tree growth. The convergence of leaf photosynthetic characteristics in distinct lineages may contribute to the persistence of species in the adjacent environment in forests or similar geographical environmental constraints. Insights into photosynthesis and plants' geographical distribution provide valuable information to investigate plant–environment interactions during their long historical evolution^[59,60]^. Molecular genetics studies have shown that lncRNAs involved in the precise control of light-mediated development. *MLNC3.2* and *MLNC4.6* are predicted as endogenous target mimic for miRNA to regulate the expression of the *SPL2*-like and *SPL33* transcription factors during light-induced anthocyanin biosynthesis and involve photosynthesis^[61]^. Thus, studying photosynthesis-associated modules would be more informative. The co-expression network analysis in *P. tomentosa* and *P. simonii* showed that genes involved in 'photosynthesis, light reaction' and 'photosynthesis' were enriched in MEbrown from *P. tomentosa* co-expression network and MEturquoise from *P. simonii* co-expression network, respectively. LncRNA expressions from accessions of the south region involving photosynthetic pathways were higher than accessions from the northern region, suggesting a conserved spatial-induced expression of lncRNAs in plants^[62,63]^. For MEbrown module in *P. tomentosa*, we discovered a lncRNA Pto_XLOC_013503 was co-expressed with *PtoMPH2*, and the expression pattern varies among geographical regions. This indicates that the effect of genes and lncRNAs may differ among geographical regions in photosynthetic efficiency and affect growth acclimation under photo-inhibitory light and fluctuating light environments^[64]^. We also found that Psi_XLOC_022416 in *P. simonii* has transcriptional regulatory relationships with *PsiLHCB7* which can be strongly expressed when light harvesting is limiting for plant growth^[65]^. *AtLHCB7* is also associated with the threshold of light-saturated photosynthesis rate and irradiance threshold for induction of photoprotective non-photochemical quenching^[66]^. Intriguingly, functional orthologs were found in homolog pairs Pto_XLOC_001831-Psi_XLOC_022416. These homologs were not found outside three Salicaceae species but were shown to have similar functions in photosynthetic pathways. We note these homologs as 'functional orthologs', which may have similar functions but have a poor ancestral relationship.

Epigenetic variation is tightly linked to environmental and fitness differences, implying its involvement in adaptive evolution^[67,68]^. In this study, *Populus* samples were well distinguished into three clusters by DNA methylation which were consistent with the origin of accessions. Interestingly, DNA methylation of accessions from the South was lower than those from the Northern accessions in both *Populus* species (Fig. 6c). Thus, DNA methylation is involved in the variation of *Populus* from different geographical regions. In photosynthetic genes, we found that *PtoLHCA1*, *PtoPnsB4,* and *PsiLHCB7* were all hypomethylated in the Southern region, demonstrating that DNA methylation may act as a regulator in plants' light harvesting process. The differentiated expression patterns of these genes across the three geographic regions (Fig. 6d, e) imply that the transcriptional regulation of photosynthetic may also undergo DNA methylation variation creating *P. tomentosa* ecotypes.

### Characterization of interspecies variation in two *Populus* species and their evolution

LncRNA works as a regulator by recruiting DNA methyltransferases or demethylases to regulate the target gene transcription. Some lncRNAs are involved in chromatin modification and RNA-directed DNA methylation (RdDM)^[69,70]^. Theoretical and empirical data showed that the stress responsiveness to fitness traits is typically an interactive modification process of genetic and epigenetic, in which epigenetic signatures are deeply interwoven with DNA sequence polymorphism^[71,72]^. Drought stress-dependent flowering vigor in the same altitudinal gradient reinforces SNP–DMC associations in adaptive evolution^[73]^. Patterns of correlation between promising selected DMRs and nearby SNPs assign causality DMRs associated with the flowering time traits and are consistent with the idea that many DMRs are the result of genetic changes for maize^[74,75]^. In this study, strong SNP-DMR correlation pairs were found when DMRs were involved in epigenetic variation between geographical regions (Fig. 6d), particularly when the co-varying SNPs were in promoter regions and protein-coding regions. Functional variants of genomic regions may have experienced strong selection pressure responsible for local adaptation within the species' widespread natural distribution^[24,76]^. We reveal distinct types of regulation between lncRNA modulators and target genes that are operative either in one species or across species^[77]^. The deregulation of Pto_XLOC_001831 expression in NE was associated with alterations in DNA methylation and genetic variation^[78]^. A missense variant and hypermethylation in the promoter region participate in the regulation of gene expression. On the contrary, the genetic locus can encode a suppressor program that is enforced by the lncRNA independent of the protein product of the locus despite the modification of DNA methylation^[79]^. DNA methylation regulates lncRNA expression to determine the dysregulation of the gene. A lncRNA arising from the *CEBPA* gene locus could compete with DNA methyltransferases, which inhibits* CEBPA* gene methylation and facilitates *CEBPA* expression^[80]^. These results provided valuable candidate allelic genes for regional breeding programs to improve photosynthetic efficiency in *Populus*.

Collectively, using two *Populus* species that contain accession from three geographical regions, our results suggest a meaningful functional role for lncRNA and DNA methylation variation in the photosynthetic convergent evolution of *Populus*. The comparison of geographical regions could inform on the adaptive potential of two closely related *Populus* species in the evolution process. However, further investigation is required to make conclusive statements concerning the evolutionary basis of DNA methylation with genetic variation. Further investigation of the mechanism underlying the recruitment of DNA methylation through lncRNAs to affect genome-wide patterns of gene regulation is warranted. Therefore, the gene editing technology of CRISPR-Cas9^[81]^ and the methylation editing technology of CRISPR-dCas9^[82]^ will help to determine the trigger for the deep-seated mechanism of naturally occurring lncRNAs and epigenetic variation and may provide a useful source of regulatory variation for tree improvement.

## SUPPLEMENTARY DATA

Supplementary data to this article can be found online.
