# Supplementary material for: LncRNA evolution and DNA methylation variation participate in photosynthesis pathways of distinct lineages of Populus
Source: For Res (Fayettev). 2023 Feb 6;3:3. doi: 10.48130/FR-2023-0003 (PMC11524286; doi:10.48130/FR-2023-0003)

Fig. S1 Box plot of regulatory roles of lncRNAs in *Populus tomentosa* and *Populus simonii.* (a-b) Comparison of the co-expressed correlations of lncRNA–PCG, neighboring PCG–PCG, random lncRNA–PCG and random PCG–PCG groups in *P. tomentosa* (a) and *P. simonii* (b). The significant differences between these groups were calculated using the Mann–Whitney U-test.

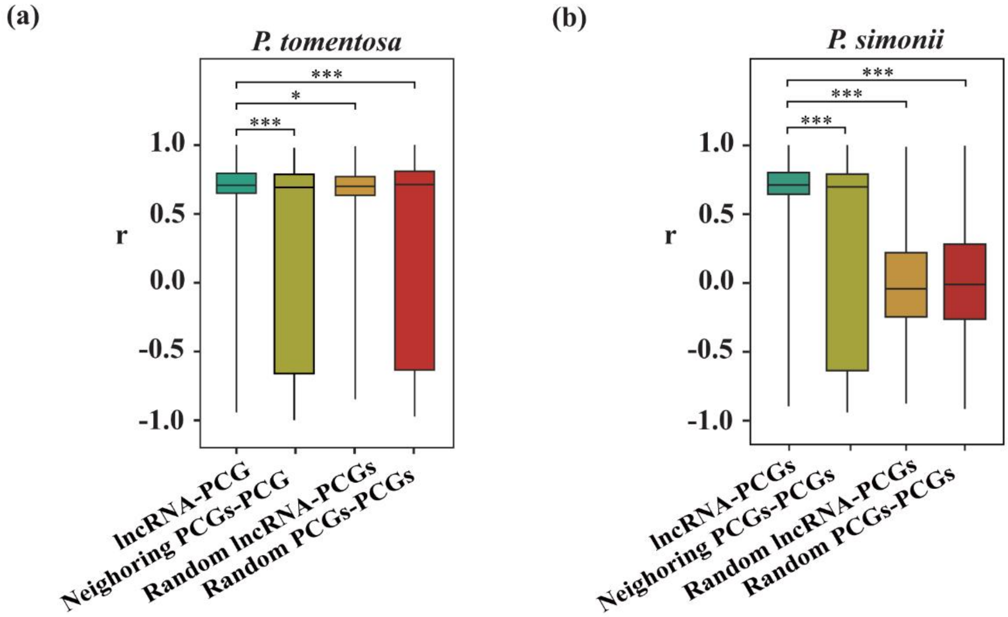

Supplement: Supplementary file 1 — Supplementary data to this article can be found online. [file FR-2023-0003-S1.zip › 10.48130_FR-2023-0003-Suppl-FigureS1.pdf]
