# Supplementary material for: LncRNA evolution and DNA methylation variation participate in photosynthesis pathways of distinct lineages of Populus
Source: For Res (Fayettev). 2023 Feb 6;3:3. doi: 10.48130/FR-2023-0003 (PMC11524286; doi:10.48130/FR-2023-0003)

Fig. S2 Gene Ontology enrichment analyses of Turquoise Module in *Populus simonii*. Circos plot shows the enrichment and differentially expressed genes in each ontology of Module Brown in *P. tomentosa*. From outer circle to inner circle is GO ontology id, number of genes and *P* -value, number of differentially expressed genes, and enrichment factors.

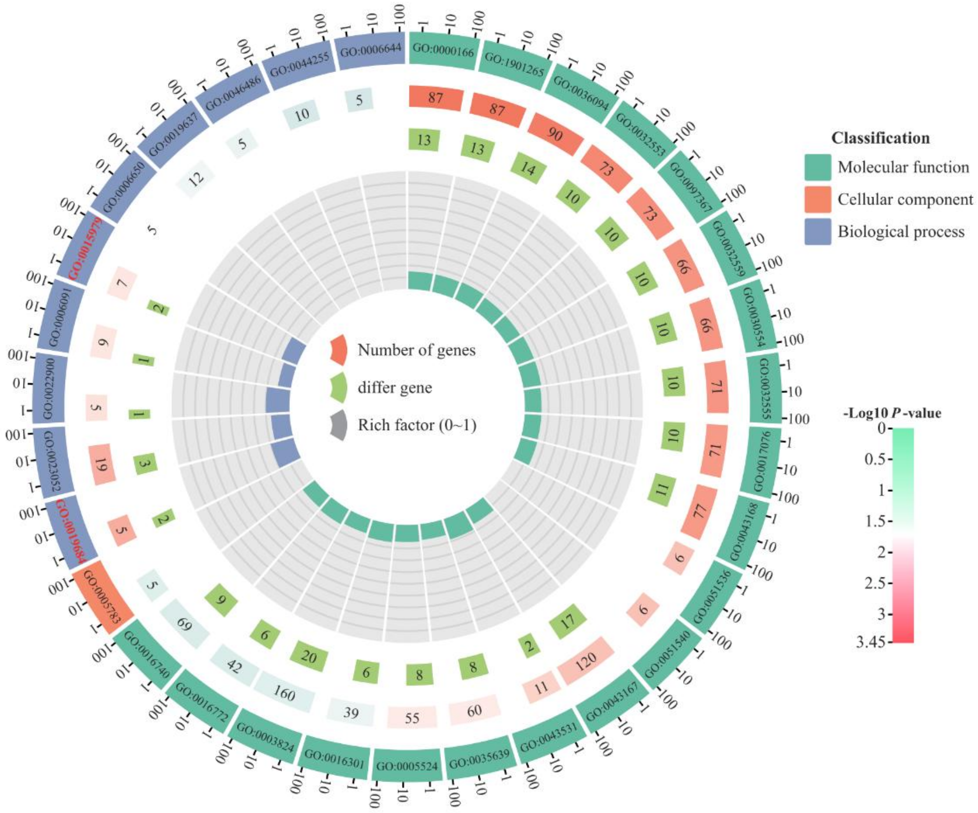

Supplement: Supplementary file 1 — Supplementary data to this article can be found online. [file FR-2023-0003-S1.zip › 10.48130_FR-2023-0003-Suppl-FigureS2.pdf]
