# Supplementary material for: LncRNA evolution and DNA methylation variation participate in photosynthesis pathways of distinct lineages of Populus
Source: For Res (Fayettev). 2023 Feb 6;3:3. doi: 10.48130/FR-2023-0003 (PMC11524286; doi:10.48130/FR-2023-0003)

Fig. S3 Geographical variation characterized by global DNA methylation patterns. (a) Hierarchical cluster maps showing global methylation relationships across all *P. tomentosa* samples from three geographical accessions. **(b)** Hierarchical cluster maps showing global methylation relationships across all *P. simonii* samples from three geographical accessions.

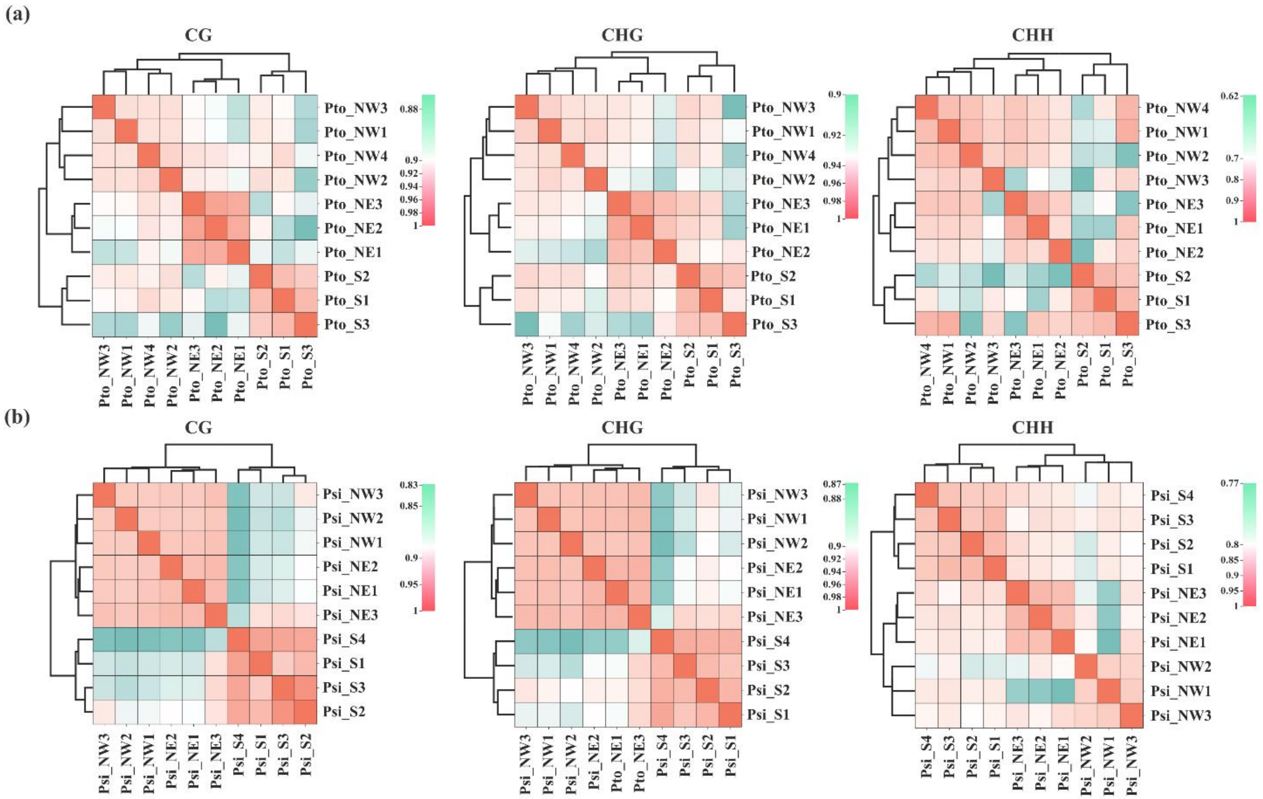

Supplement: Supplementary file 1 — Supplementary data to this article can be found online. [file FR-2023-0003-S1.zip › 10.48130_FR-2023-0003-Suppl-FigureS3.pdf]
