# Supplementary material for: LncRNA evolution and DNA methylation variation participate in photosynthesis pathways of distinct lineages of Populus
Source: For Res (Fayettev). 2023 Feb 6;3:3. doi: 10.48130/FR-2023-0003 (PMC11524286; doi:10.48130/FR-2023-0003)

Fig. S4 The distribution of differentially methylated genes in *Populus tomentosa* and *Populus simonii*. (a) Percentage distribution of differentially methylated genes in genomic features in *Populus tomentosa* and *Populus simonii*. **(b)** Overlaps between lncRNAs-target PCGs and differentially methylated genes in *P. tomentosa* and *P. simonii*. **(c)** Overlaps between differentially methylated lncRNAs-target PCGs and differentially expressed genes in *P. tomentosa* and *P. simonii*.

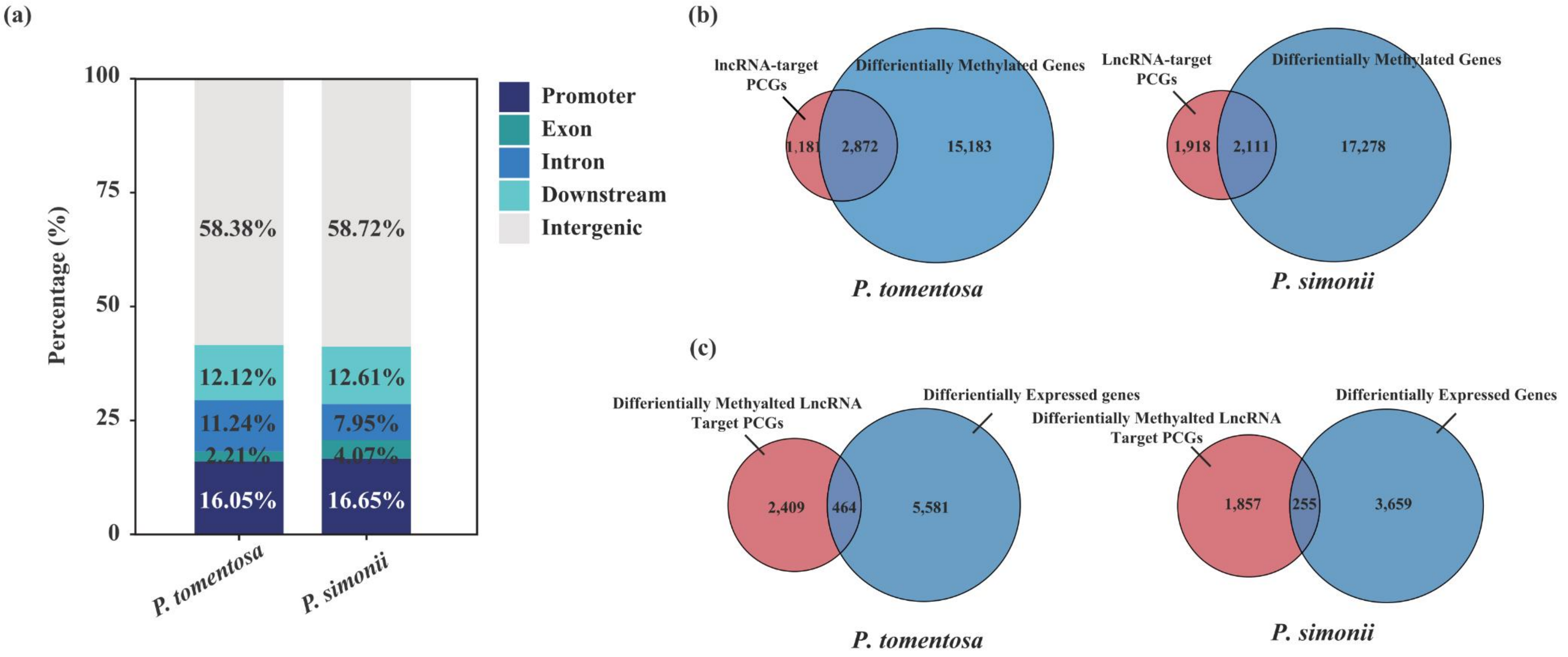

Supplement: Supplementary file 1 — Supplementary data to this article can be found online. [file FR-2023-0003-S1.zip › 10.48130_FR-2023-0003-Suppl-FigureS4.pdf]
