# Supplementary material for: LncRNA evolution and DNA methylation variation participate in photosynthesis pathways of distinct lineages of Populus
Source: For Res (Fayettev). 2023 Feb 6;3:3. doi: 10.48130/FR-2023-0003 (PMC11524286; doi:10.48130/FR-2023-0003)

Fig. S5 Differentially methylated regions (DMRs) correlated with genetic variation. (a-b) Scatter plots of *P. tomentosa* (a) and *P. simonii* (b) of SNPs distance with DMRs, and the relation of the one-sided permutation test between *P*- value and r^2. Red dots represent SNPs fulfilling the significant selection between each pairwise DMR-SNP.

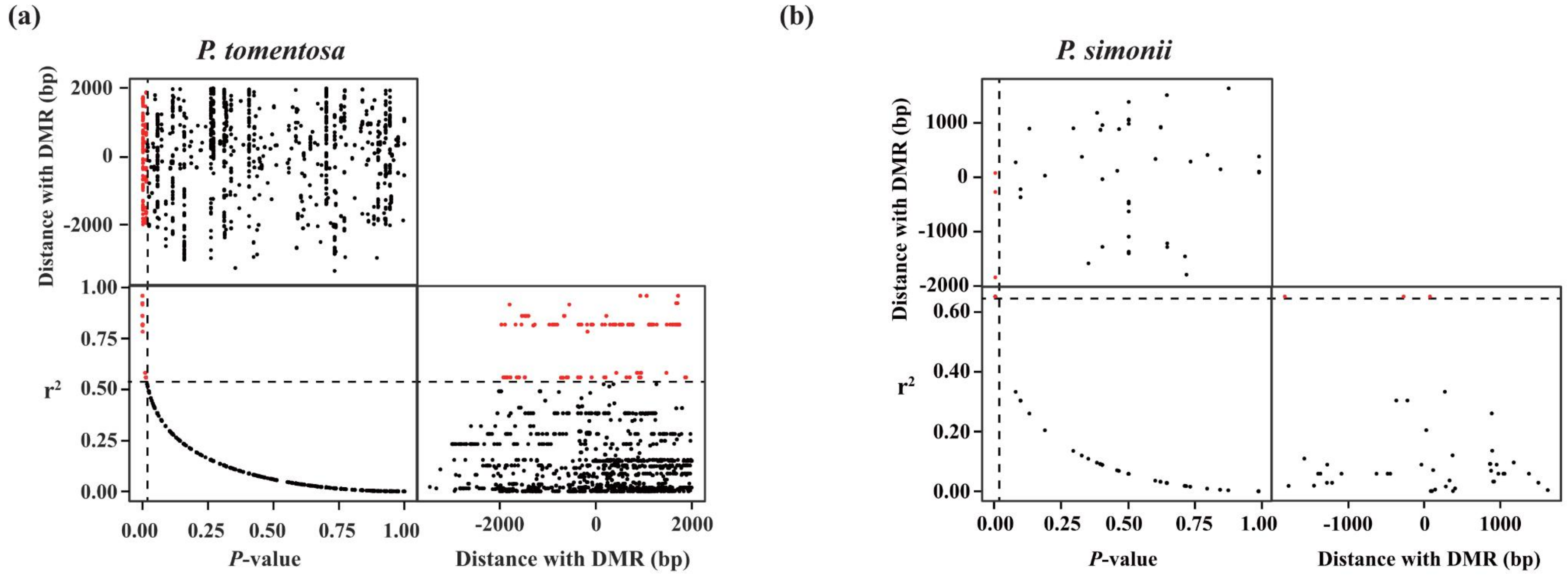

Supplement: Supplementary file 1 — Supplementary data to this article can be found online. [file FR-2023-0003-S1.zip › 10.48130_FR-2023-0003-Suppl-FigureS5.pdf]
