# Supplementary material for: LncRNA evolution and DNA methylation variation participate in photosynthesis pathways of distinct lineages of Populus
Source: For Res (Fayettev). 2023 Feb 6;3:3. doi: 10.48130/FR-2023-0003 (PMC11524286; doi:10.48130/FR-2023-0003)
Supplement: Supplementary file 1 — Supplementary data to this article can be found online. [file FR-2023-0003-S1.zip › 10.48130_FR-2023-0003-Suppl-FigureS6.pdf]

Method S2 The process to calculate DMRs.

We employed a sliding-window approach with a window size of 100 bp and step length of 50 bp to calculate DMRs. Windows with at least five effective cytosines were retained. DNA methylation levels were compared pairwise with Fisher's exact test, and the *P*-values were adjusted for multiple comparisons using the Benjamini–Hochberg method. Windows with FDR <0.01 were selected, and regions with an absolute methylation level difference of 0.4, 0.2, and 0.1 for CG, CHG, and CHH, respectively, were retained for subsequent analysis. The adjacent windows within 100 bp of each other were merged as the final DMRs.
