# Supplementary material for: LncRNA evolution and DNA methylation variation participate in photosynthesis pathways of distinct lineages of Populus
Source: For Res (Fayettev). 2023 Feb 6;3:3. doi: 10.48130/FR-2023-0003 (PMC11524286; doi:10.48130/FR-2023-0003)
Supplement: Supplementary file 1 — Supplementary data to this article can be found online. [file FR-2023-0003-S1.zip › 10.48130_FR-2023-0003-Suppl-TableS1.pdf]

Table S1 Phenotypic variation among ten accessions from S, NW and NE climate regions in *Populus tomentosa* and *Populus simonii*.

Species	Region	S[1]	NW[2]	NE[3]	S vs. NW[4]	S vs. NE[5]	NW vs. NE[6]	Coefficient of Variation
Populus tomentosa	Pn	23.53±0.43	19.11±0.64	18.10±0.37	1.00E-03	0.00E+00	1.90E-01	0.29
	Tr	6.67±0.60	12.57±1.03	8.53±0.37	2.00E-03	1.47E-01	2.10E-02	0.13
	WUE	3.64±0.43	1.71±0.10	2.11±0.02	1.00E-03	3.00E-03	2.51E-01	0.34
	Gs	0.42±0.04	0.44±0.01	0.57±0.02	4.90E-02	6.00E-03	1.26E-01	0.09
	Ci	253.15±18.33	279.32±5.50	313.52±1.52	3.43E-01	3.70E-02	1.28E-01	0.11
Populus simonii	Pn	21.19±0.43	17.8±0.51	16.05±0.51	2.00E-03	0.00E+00	4.10E-02	0.13
	Tr	9.25±0.25	8.42±0.42	9.63±0.08	7.00E-03	1.32E-01	1.00E-03	0.06
	WUE	2.29±0.05	2.12±0.12	1.66±0.06	1.77E-01	1.00E-03	6.00E-03	0.15
	Gs	0.62±0.01	0.49±0.002	0.58±0.01	0.00E+00	1.10E-02	0.00E+00	0.11
	Ci	304.69±0.52	323.79±1.26	318.48±0.17	0.00E+00	0.00E+00	2.00E-03	0.03

[1,2,3] Mean±SE

[4,5,6] *P*-value of one-way ANOVA followed by POST HOC LSD
