# Supplementary material for: LncRNA evolution and DNA methylation variation participate in photosynthesis pathways of distinct lineages of Populus
Source: For Res (Fayettev). 2023 Feb 6;3:3. doi: 10.48130/FR-2023-0003 (PMC11524286; doi:10.48130/FR-2023-0003)
Supplement: Supplementary file 1 — Supplementary data to this article can be found online. [file FR-2023-0003-S1.zip › 10.48130_FR-2023-0003-Suppl-TableS10.pdf]

Table S10 Interacting DMR and SNP in 2kb.

DMR-ID	*P*-value	r^2	Gene_id	Name	Feature	SNP ID
PtoPnsB4-DMR3	7.37E-02	0.35	Ptom.012G00615	*PtoPnsB4*	Downstream	Chr12-7793518
PtoPnsB4-DMR3	7.37E-02	0.35	Ptom.012G00615	*PtoPnsB4*	Downstream	Chr12-7794411
PtoPnsB4-DMR3	7.37E-02	0.35	Ptom.012G00615	*PtoPnsB4*	Downstream	Chr12-7793518
PtoPnsB4-DMR3	7.37E-02	0.35	Ptom.012G00615	*PtoPnsB4*	Downstream	Chr12-7794411
PtoPnsB4-DMR2	7.13E-02	0.35	Ptom.012G00615	*PtoPnsB4*	Downstream	Chr12-7793463
PtoPnsB4-DMR2	7.13E-02	0.35	Ptom.012G00615	*PtoPnsB4*	Downstream	Chr12-7793652
PtoPnsB4-DMR2	7.13E-02	0.35	Ptom.012G00615	*PtoPnsB4*	Downstream	Chr12-7794094
PtoPnsB4-DMR2	7.13E-02	0.35	Ptom.012G00615	*PtoPnsB4*	Downstream	Chr12-7794388
PtoPnsB4-DMR2	7.13E-02	0.35	Ptom.012G00615	*PtoPnsB4*	Downstream	Chr12-7793961
PtoPnsB4-DMR2	5.75E-02	0.38	Ptom.012G00615	*PtoPnsB4*	Downstream	Chr12-7793178
PtoPnsB4-DMR2	5.75E-02	0.38	Ptom.012G00615	*PtoPnsB4*	Downstream	Chr12-7793188
PtoPnsB4-DMR2	5.75E-02	0.38	Ptom.012G00615	*PtoPnsB4*	Downstream	Chr12-7793983
PtoPnsB4-DMR2	5.75E-02	0.38	Ptom.012G00615	*PtoPnsB4*	Downstream	Chr12-7793992
PtoPnsB4-DMR2	5.75E-02	0.38	Ptom.012G00615	*PtoPnsB4*	Downstream	Chr12-7794088
PtoPnsB4-DMR2	5.75E-02	0.38	Ptom.012G00615	*PtoPnsB4*	Downstream	Chr12-7794170
PtoPnsB4-DMR2	5.75E-02	0.38	Ptom.012G00615	*PtoPnsB4*	Downstream	Chr12-7794180
PtoPnsB4-DMR2	5.75E-02	0.38	Ptom.012G00615	*PtoPnsB4*	Downstream	Chr12-7794225
PtoPnsB4-DMR3	5.62E-02	0.38	Ptom.012G00615	*PtoPnsB4*	Exon	Chr12-7791606
PtoPnsB4-DMR3	5.62E-02	0.38	Ptom.012G00615	*PtoPnsB4*	Exon	Chr12-7791882
PtoPnsB4-DMR3	5.62E-02	0.38	Ptom.012G00615	*PtoPnsB4*	Exon	Chr12-7792185
PtoPnsB4-DMR3	5.62E-02	0.38	Ptom.012G00615	*PtoPnsB4*	Exon	Chr12-7792247
PtoPnsB4-DMR3	5.62E-02	0.38	Ptom.012G00615	*PtoPnsB4*	Exon	Chr12-7792258
PtoPnsB4-DMR3	5.62E-02	0.38	Ptom.012G00615	*PtoPnsB4*	Exon	Chr12-7792303
PtoPnsB4-DMR3	5.62E-02	0.38	Ptom.012G00615	*PtoPnsB4*	Exon	Chr12-7792349

PtoPnsB4-DMR1	7.53E-07	0.96	Ptom.012G00615	*PtoPnsB4*	Exon	Chr12-7790159
PtoPnsB4-DMR1	7.53E-07	0.96	Ptom.012G00615	*PtoPnsB4*	Intron	Chr12-7790812
PsiPSBR-DMR1	4.73E-03	0.65	Potri.001G438700	*PsiPSBR*	Intron	Chr1-46474560
PsiPSBR-DMR1	4.73E-03	0.65	Potri.001G438700	*PsiPSBR*	Promoter	Chr1-46476479
