# Supplementary material for: LncRNA evolution and DNA methylation variation participate in photosynthesis pathways of distinct lineages of Populus
Source: For Res (Fayettev). 2023 Feb 6;3:3. doi: 10.48130/FR-2023-0003 (PMC11524286; doi:10.48130/FR-2023-0003)
Supplement: Supplementary file 1 — Supplementary data to this article can be found online. [file FR-2023-0003-S1.zip › 10.48130_FR-2023-0003-Suppl-TableS11.pdf]

Supporting Methods

Method S1 The process of RNA-sequencing.

The RNase-free DNase set (Qiagen) was used three times for DNA digestion during the RNA purification. Illumina TruSeq® Stranded Total RNA Library Prep Plant were used to remove Ribosome RNA (rRNA). NanoDrop ND-1000 and Agilent Bioanalyzer 2100 were used to assess the quality of the RNA samples before the construction of strand-specific RNA-seq libraries. After being quantified by a Qubit 2.0 Fluorometer and Agilent 2100 bioanalyzer, the strand-specific RNA-seq libraries were sequenced on the Illumina HiSeq X Ten platform. Library construction and sequencing were implemented by NovoGene Co., Ltd (Beijing, China). Paired-end RNA-seq reads were obtained for further analysis. Reads containing adapters reads, poly-N sequences whose number accounted for more than 5% of the total (quality score, Q ⩾ 30), and low-quality reads were removed using Trimmomatic (Bolger *et al.*, 2014).

Reference:

Bolger, A.M., Lohse, M. and Usadel, B. (2014) Trimmomatic: a flexible trimmer for Illumina sequence data. *Bioinformatics*, 30, 2114–2120.

Method S2 The process to calculate DMRs.

We employed a sliding-window approach with a window size of 100 bp and step length of 50 bp to calculate DMRs. Windows with at least five effective cytosines were retained. DNA methylation levels were compared pairwise with Fisher's exact test, and the *P*-values were adjusted for multiple comparisons using the Benjamini–Hochberg method. Windows with FDR <0.01 were selected, and regions with an absolute methylation level difference of 0.4, 0.2, and 0.1 for CG, CHG, and CHH, respectively, were retained for subsequent analysis. The adjacent windows within 100 bp of each other were merged as the final DMRs.
