# Supplementary material for: LncRNA evolution and DNA methylation variation participate in photosynthesis pathways of distinct lineages of Populus
Source: For Res (Fayettev). 2023 Feb 6;3:3. doi: 10.48130/FR-2023-0003 (PMC11524286; doi:10.48130/FR-2023-0003)
Supplement: Supplementary file 1 — Supplementary data to this article can be found online. [file FR-2023-0003-S1.zip › 10.48130_FR-2023-0003-Suppl-TableS2.pdf]

Table S2 RNA-sequencing data statistics.

Species	Accessions ID	Climate region	Total reads	Mapping Rate (%)
Populus tomentosa	Pto_S1	Southern	27,397,209	84.76
	Pto_S2	Southern	26,603,614	84.67
	Pto_S3	Southern	26,059,934	84.67
	Pto_NW1	Northwestern	29,521,433	85.93
	Pto_NW2	Northwestern	24,542,716	86.22
	Pto_NW3	Northwestern	32,479,888	84.68
	Pto_NW4	Northwestern	26,382,112	86
	Pto_NE1	Northeastern	30,760,816	86.76
	Pto_NE2	Northeastern	28,096,305	86.97
	Pto_NE3	Northeastern	23,682,918	84.05
Populus simonii	Psi_S1	Southern	34,320,111	89.95
	Psi_S2	Southern	26,897,429	90.04
	Psi_S3	Southern	28,472,155	90
	Psi_S4	Southern	31,781,682	90.76
	Psi_NW1	Northwestern	28,919,374	90.71
	Psi_NW2	Northwestern	28,917,215	90.75
	Psi_NW3	Northwestern	30,458,687	90.39
	Psi_NE1	Northeastern	30,658,434	89.2
	Psi_NE2	Northeastern	26,712,015	90.9
	Psi_NE3	Northeastern	30,458,143	90.9
