# Supplementary material for: LncRNA evolution and DNA methylation variation participate in photosynthesis pathways of distinct lineages of Populus
Source: For Res (Fayettev). 2023 Feb 6;3:3. doi: 10.48130/FR-2023-0003 (PMC11524286; doi:10.48130/FR-2023-0003)
Supplement: Supplementary file 1 — Supplementary data to this article can be found online. [file FR-2023-0003-S1.zip › 10.48130_FR-2023-0003-Suppl-TableS3.pdf]

Table S3 The number of correlations between lncRNAs and PCGs.

Species	Significant lncRNA-PCG				Significant random lncRNA-PCG				Significant random PCG-PCG			
	Pairs	r>0	r<0	ABS |r>0.8|[1]	Pairs	r>0	r<0	ABS |r>0.8|[2]	Pairs	r>0	r<0	ABS |r>0.8|[3]
Populus tomentosa	686	558	127	177	241	182	59	46	395	242	153	93
Populus simonii	452	362	90	119	249	170	79	63	498	289	209	112

[123] Extremely positive correlations between members of lncRNA–PCG, random lncRNA-PCG, and random PCG-PCG
