# Supplementary material for: LncRNA evolution and DNA methylation variation participate in photosynthesis pathways of distinct lineages of Populus
Source: For Res (Fayettev). 2023 Feb 6;3:3. doi: 10.48130/FR-2023-0003 (PMC11524286; doi:10.48130/FR-2023-0003)
Supplement: Supplementary file 1 — Supplementary data to this article can be found online. [file FR-2023-0003-S1.zip › 10.48130_FR-2023-0003-Suppl-TableS4.pdf]

Table S4 Differentially expressed genes and lncRNAs in photosynthetic modules of *Populus tomentosa* and *Populus simonii*.

Gene_id	Name	Log2FC	adjust *P*-value	Comparison	Function
Potri.001G438700	*PsiPSBR*	-2.70	1.95E-08	S vs. NW	photosystem II subunit R
Potri.001G438700	*PsiPSBR*	-2.29	1.36E-08	S vs. NE	photosystem II subunit R
Potri.005G095900	*PsiKUP1*	-2.15	3.32E-10	S vs. NW	Potassium ion transporter activity
Potri.005G095900	*PsiKUP1*	-2.26	9.33E-11	S vs. NE	Potassium ion transporter activity
Potri.005G258600	*PsiLHCB7*	-2.08	5.14E-54	S vs. NW	Chlorophyll A-B binding family protein
Potri.005G258600	*PsiLHCB7*	-2.99	9.05E-05	S vs. NE	Chlorophyll A-B binding family protein
Potri.007G061400	*PsiNIP2*	-1.36	6.63E-03	S vs. NW	NEP-interacting protein 2
Potri.007G061400	*PsiNIP2*	-1.27	1.03E-05	S vs. NE	NEP-interacting protein 2
Psi_XLOC_011671	Psi_XLOC_011671	-2.33	6.57E-03	S vs. NW	Target *Potri.007G061400*
Psi_XLOC_011671	Psi_XLOC_011671	-2.46	1.43E-03	S vs. NE	Target *Potri.007G061400*
Psi_XLOC_022416	Psi_XLOC_022416	-2.56	2.46E-02	S vs. NW	Target *Potri.005G258600* and *Potri.001G438700*
Psi_XLOC_022416	Psi_XLOC_022416	-2.80	1.32E-02	S vs. NE	Target *Potri.005G258600* and *Potri.001G438700*
Psi_XLOC_022701	Psi_XLOC_022701	-3.08	2.30E-05	S vs. NW	Target *Potri.005G095900*
Psi_XLOC_022701	Psi_XLOC_022701	-4.07	1.87E-18	S vs. NE	Target *Potri.005G095900*
Pto_XLOC_001831	Pto_XLOC_001831	-2.48	4.12E-07	S vs. NE	Target *Ptom.012G00615*
Pto_XLOC_010062	Pto_XLOC_010062	-5.95	2.21E-16	S vs. NE	Target *Ptom.012G000615*
Pto_XLOC_013503	Pto_XLOC_013503	-2.61	3.97E-17	S vs. NW	Target *Ptom.018G001293*
Pto_XLOC_013503	Pto_XLOC_013503	-2.02	3.92E-10	S vs. NE	Target *Ptom.018G001293*
Pto_XLOC_026190	Pto_XLOC_026190	-1.42	3.98E-05	S vs. NW	Target *Ptom.010.01955*
Pto_XLOC_026190	Pto_XLOC_026190	-4.71	3.81E-36	S vs. NE	Target *Ptom.010.01955*
Ptom.010G.01955	*PtoPPL1*	-1.24	7.40E-10	S vs. NW	PsbP-like protein 1
Ptom.010G.01955	*PtoPPL1*	-1.85	2.06E-12	S vs. NE	PsbP-like protein 1
Ptom.010G.00307	*PtoLHCA1*	-1.78	1.62E-05	S vs. NE	Photosystem I light harvesting complex gene 1
Ptom.012G00615	*PtoPnsB4*	-1.07	1.13E-07	S vs. NW	NDH dependent flow 6

Ptom.012G00615	*PtoPnsB4*	-1.31	3.40E-08	S vs. NE	NDH dependent flow 6
Ptom.018G01293	*PtoMPH2*	-2.01	1.68E-09	S vs. NW	Chloroplast thylakoid lumen protein
Ptom.018G01293	*PtoMPH2*	-2.18	4.42E-10	S vs. NE	Chloroplast thylakoid lumen protein
