# Supplementary material for: LncRNA evolution and DNA methylation variation participate in photosynthesis pathways of distinct lineages of Populus
Source: For Res (Fayettev). 2023 Feb 6;3:3. doi: 10.48130/FR-2023-0003 (PMC11524286; doi:10.48130/FR-2023-0003)
Supplement: Supplementary file 1 — Supplementary data to this article can be found online. [file FR-2023-0003-S1.zip › 10.48130_FR-2023-0003-Suppl-TableS5.pdf]

Table S5 Methylome data bisulfite conversion rate and sequencing statistics.

Species	Accessions ID	Climate region	Total reads	Mapping Rate (%)	Duplication Rate (%)	Conversion rate%	Raw Bases (Gb)	Clean Bases (Gb)	GC Content (%)
Populus tomentosa	Pto_S1	Southern	53,432,734	61.6	14.56	99.90	16.36	14.52	20.78
	Pto_S2	Southern	52,662,445	56.6	22.62	99.88	16.14	14.31	28.83
	Pto_S3	Southern	67,405,304	62	17.65	99.92	20.52	18.42	21.32
	Pto_NW1	Northwestern	56,386,528	63.3	16.47	99.89	17.29	15.31	20.5
	Pto_NW2	Northwestern	61,111,657	64.3	15.73	99.88	18.58	16.72	20.49
	Pto_NW3	Northwestern	50,609,159	65.7	15.2	99.90	15.47	13.77	21.58
	Pto_NW4	Northwestern	54,509,052	64.2	18.51	99.90	16.68	14.83	22.96
	Pto_NE1	Northeastern	52,214,880	64.3	22.82	99.91	15.92	14.26	26.68
	Pto_NE2	Northeastern	60,198,127	66.8	15.14	99.89	18.33	16.44	21.23
	Pto_NE3	Northeastern	61,442,701	61.1	14.04	99.88	18.72	16.76	21.95
Populus simonii	Psi_S1	Southern	64,970,011	50.9	16.15	99.86	19.68	17.89	19.28
	Psi_S2	Southern	46,705,422	53.7	14.2	99.87	14.17	12.81	19.66
	Psi_S3	Southern	67,937,505	51.9	16.75	99.86	20.6	18.69	19.4
	Psi_S4	Southern	57,270,814	54.3	16.15	99.85	17.38	15.76	18.88
	Psi_NW1	Northwestern	71,174,382	49.5	19.27	99.85	21.62	19.56	18.25
	Psi_NW2	Northwestern	49,234,084	50.8	17.52	99.83	14.97	13.44	17.94
	Psi_NW3	Northwestern	48,692,627	49	16.61	99.85	14.79	13.34	17.69
	Psi_NE1	Northeastern	53,162,940	51.9	13.64	99.87	16.17	14.51	19.35
	Psi_NE2	Northeastern	56,170,820	54.6	16.59	99.88	17.01	15.47	20.16
	Psi_NE3	Northeastern	72,340,809	47.6	19.6	99.86	21.94	19.89	18.72
