# Supplementary material for: LncRNA evolution and DNA methylation variation participate in photosynthesis pathways of distinct lineages of Populus
Source: For Res (Fayettev). 2023 Feb 6;3:3. doi: 10.48130/FR-2023-0003 (PMC11524286; doi:10.48130/FR-2023-0003)
Supplement: Supplementary file 1 — Supplementary data to this article can be found online. [file FR-2023-0003-S1.zip › 10.48130_FR-2023-0003-Suppl-TableS6.pdf]

Table S6 Data description of methylation level for ten *Populus tomentosa* and *Populus simonii* accessions.

Accession ID	Accession ID	mCG/CG (%)	mCHG/CHG (%)	mCHH/CHH (%)	Coefficient of Variation of CG	Coefficient of Variation of CHG	Coefficient of Variation of CHH
Populus tomentosa	Pto_S1	58.50	38.30	2.60	0.12	0.19	0.26
	Pto_S2	80.90	60.20	4.90			
	Pto_S3	61.70	42.30	3.00			
	Pto_NW1	63.40	45.60	4.10			
	Pto_NW2	63.00	43.00	3.10			
	Pto_NW3	63.70	42.70	2.50			
	Pto_NW4	73.50	55.00	4.00			
	Pto_NE1	77.10	60.70	5.30			
	Pto_NE2	62.40	44.20	4.60			
	Pto_NE3	58.90	36.50	3.40			
Populus simonii	Psi_S1	41.20	33.10	4.60	0.05	0.06	0.12
	Psi_S2	37.50	29.20	4.20			
	Psi_S3	39.30	31.00	5.00			
	Psi_S4	46.40	37.90	5.60			
	Psi_NW1	37.80	32.30	4.80			
	Psi_NW2	41.90	33.90	5.40			
	Psi_NW3	39.00	30.70	4.60			
	Psi_NE1	39.40	31.20	4.10			
	Psi_NE2	38.50	30.40	4.30			
	Psi_NE3	42.40	35.80	5.50			
