# Supplementary material for: LncRNA evolution and DNA methylation variation participate in photosynthesis pathways of distinct lineages of Populus
Source: For Res (Fayettev). 2023 Feb 6;3:3. doi: 10.48130/FR-2023-0003 (PMC11524286; doi:10.48130/FR-2023-0003)
Supplement: Supplementary file 1 — Supplementary data to this article can be found online. [file FR-2023-0003-S1.zip › 10.48130_FR-2023-0003-Suppl-TableS7.pdf]

Table S7 The number of DMRs in genomic features in *Populus tomentosa* and *Populus simonii*.

Species	Comparison	Context	Promoter	Exon	Intron	downstream	Intergenic
Populus tomentosa	Southern vs. Northwestern	CG	2081 (15.82%)	681 (5.17%)	2535 (19.27%)	1752 (13.32%)	6102 (46.39%)
		CHG	5473 (15.7%)	369 (1.05%)	3011 (8.63%)	4068 (11.67%)	21937 (62.93%)
		CHH	1149 (18.52%)	63 (1.01%)	401 (6.46%)	797 (12.84%)	3794 (61.15%)
	Southern vs. Northeastern	CG	2169 (15.79%)	808 (5.88%)	2502 (18.22%)	1780 (12.96%)	6470 (47.12%)
		CHG	5263 (14.45%)	533 (1.46%)	3542 (9.72%)	4082 (11.21%)	22986 (63.13%)
		CHH	2490 (18.05%)	116 (0.84%)	940 (6.81%)	1552 (11.25%)	8693 (63.03%)
	Northwestern vs. Northeastern	CG	1403 (17.79%)	442 (5.6%)	1425 (18.06%)	1133 (14.36%)	3483 (44.16%)
		CHG	2612 (16%)	235 (1.44%)	1957 (11.99%)	2012 (12.33%)	9499 (58.22%)
		CHH	1208 (19.39%)	34 (0.54%)	381 (6.11%)	825 (13.24%)	3779 (60.68%)
Populus simonii	Southern vs. Northwestern	CG	1013 (15.87%)	640 (10.02%)	1104 (17.29%)	809 (12.67%)	2816 (44.12%)
		CHG	4810 (16.34%)	663 (2.25%)	1710 (5.81%)	3611 (12.27%)	18625 (63.3%)
		CHH	785 (19.59%)	98 (2.44%)	135 (3.36%)	567 (14.15%)	2421 (60.43%)
	Southern vs. Northeastern	CG	1774 (15.46%)	1423 (12.4%)	2257 (19.68%)	1419 (12.37%)	4595 (40.06%)
		CHG	5826 (16.54%)	1079 (3.06%)	2240 (6.36%)	4394 (12.47%)	21677 (61.55%)
		CHH	1440 (18.12%)	181 (2.27%)	294 (3.7%)	1011 (12.72%)	5017 (63.16%)
	Northwestern vs. Northeastern	CG	223 (18.69%)	114 (9.55%)	240 (20.11%)	174 (14.58%)	442 (37.04%)
		CHG	3004 (16.64%)	463 (2.56%)	1153 (6.38%)	2322 (12.86%)	11109 (61.54%)
		CHH	373 (19.35%)	43 (2.23%)	60 (3.11%)	273 (14.16%)	1178 (61.13%)
