# Supplementary material for: LncRNA evolution and DNA methylation variation participate in photosynthesis pathways of distinct lineages of Populus
Source: For Res (Fayettev). 2023 Feb 6;3:3. doi: 10.48130/FR-2023-0003 (PMC11524286; doi:10.48130/FR-2023-0003)
Supplement: Supplementary file 1 — Supplementary data to this article can be found online. [file FR-2023-0003-S1.zip › 10.48130_FR-2023-0003-Suppl-TableS8.pdf]

Table S8 Differentially methylated regions in photosynthetic hubgenes.

Comparison	Chr	Start	End	Context	Fold change	Regulated	DMR ID	DMR-associated Genes	Genomic Feature
S vs. NW	Chr10	18002801	18002950	CHH	28.02	hyper	*PtoLHCA1*-DMR1	*PtoLHCA1*	Promoter
S vs. NE	Chr10	18002801	18002950	CHH	28.02	hyper	*PtoLHCA1*-DMR1	*PtoLHCA1*	Promoter
S vs. NW	Chr12	7788951	7789100	CG	50.11	hyper	*PtoPnsB4*-DMR1	*PtoPnsB4*	Promoter
S vs. NE	Chr12	7788951	7789100	CG	55.71	hyper	*PtoPnsB4*-DMR1	*PtoPnsB4*	Promoter
S vs. NE	Chr12	7793051	7793150	CG	53.33	hyper	*PtoPnsB4*-DMR2	*PtoPnsB4*	Downstream
S vs. NW	Chr12	7793501	7793650	CHG	35.27	hyper	*PtoPnsB4*-DMR3	*PtoPnsB4*	Downstream
S vs. NE	Chr12	7793501	7793650	CHG	33.08	hyper	*PtoPnsB4*-DMR3	*PtoPnsB4*	Downstream
S vs. NE	Chr10	18002001	18003500	CHH	22.61	hyper	*PtoLHCA1*-DMR1	*PtoLHCA1*	Promoter
S vs. NW	Chr18	13397701	13397950	CHH	10.68	hyper	*PtoMPH1*-DMR1	*PtoMPH1*	Promoter
NW vs. NE	Chr18	13398851	13398950	CHH	15.91	hyper	*PtoMPH1*-DMR2	*PtoMPH1*	Promoter
S vs. NW	Chr1	46476401	46476550	CG	43.86	hyper	*PsiPSBR*-DMR1	*PsiPSBR*	Promoter
S vs. NW	Chr1	46477151	46477250	CHH	-16.15	hypo	*PsiPSBR*-DMR2	*PsiPSBR*	Promoter
