# Supplementary material for: LncRNA evolution and DNA methylation variation participate in photosynthesis pathways of distinct lineages of Populus
Source: For Res (Fayettev). 2023 Feb 6;3:3. doi: 10.48130/FR-2023-0003 (PMC11524286; doi:10.48130/FR-2023-0003)
Supplement: Supplementary file 1 — Supplementary data to this article can be found online. [file FR-2023-0003-S1.zip › 10.48130_FR-2023-0003-Suppl-TableS9.pdf]

**Table S9 Correlation analysis between differentially methylated region and local SNP in *Populus tomentosa* and *Populus simonii*.**

| Species | Comparison | Context | Type | DMR | Interacting SNP in LD |
|---|---|---|---|---|---|
| *Populus tomentosa* | Southern vs. Northwestern | CG | Hypo in Northwestern | 0 | / |
| | | | Hyper in Northwestern | 1 | 12 |
| | | CHG | Hypo in Northwestern | 0 | / |
| | | | Hyper in Northwestern | 1 | 4 |
| | | CHH | Hypo in Northwestern | 0 | / |
| | | | Hyper in Northwestern | 0 | 1 |
| | Southern vs. Northeastern | CG | Hypo in Northeastern | 0 | / |
| | | | Hyper in Northeastern | 2 | 70 |
| | | CHG | Hypo in Northeastern | 0 | / |
| | | | Hyper in Northeastern | 1 | 4 |
| | | CHH | Hypo in Northeastern | 0 | / |
| | | | Hyper in Northeastern | 2 | 0 |
| | Northwestern vs. Northeastern | CG | Hypo in Northeastern | 0 | / |
| | | | Hyper in Northeastern | 0 | / |
| | | CHG | Hypo in Northeastern | 0 | / |
| | | | Hyper in Northeastern | 0 | / |
| | | CHH | Hypo in Northeastern | 0 | / |
| | | | Hyper in Northeastern | 1 | 1 |
| *Populus simonii* | Southern vs. Northwestern | CG | Hypo in Northwestern | 0 | / |
| | | | Hyper in Northwestern | 0 | / |
| | | CHG | Hypo in Northwestern | 0 | / |
| | | | Hyper in Northwestern | 0 | / |
| | | CHH | Hypo in Northwestern | 0 | / |

| | | | | | |
|---|---|---|---|---|---|
| | | | Hyper in Northwestern | 0 | / |
| | Southern vs. Northeastern | CG | Hypo in Northeastern | 0 | / |
| | | | Hyper in Northeastern | 1 | 2 |
| | | CHG | Hypo in Northeastern | 0 | / |
| | | | Hyper in Northeastern | 0 | / |
| | | CHH | Hypo in Northeastern | 0 | / |
| | | | Hyper in Northeastern | 0 | / |
| | Northwestern vs. Northeastern | CG | Hypo in Northeastern | 0 | / |
| | | | Hyper in Northeastern | 0 | / |
| | | CHG | Hypo in Northeastern | 0 | / |
| | | | Hyper in Northeastern | 0 | / |
| | | CHH | Hypo in Northeastern | 0 | / |
| | | | Hyper in Northeastern | 0 | / |
